# Centromeric cohesion failure invokes a conserved choreography of chromosomal mis-segregations in pancreatic neuroendocrine tumor

**DOI:** 10.1186/s13073-020-00730-9

**Published:** 2020-04-28

**Authors:** Rene Quevedo, Anna Spreafico, Jeff Bruce, Arnavaz Danesh, Samah El Ghamrasni, Amanda Giesler, Youstina Hanna, Cherry Have, Tiantian Li, S. Y. Cindy Yang, Tong Zhang, Sylvia L. Asa, Benjamin Haibe-Kains, Monika Krzyzanowska, Adam C. Smith, Simron Singh, Lillian L. Siu, Trevor J. Pugh

**Affiliations:** 1 Princess Margaret Cancer Centre, University Health Network, 610 University Avenue, Suite 5-718, Toronto, Ontario M5G 2M9 Canada; 2 Department of Medical Biophysics, University of Toronto, Toronto, Ontario Canada; 3 Division of Medical Oncology and Hematology, University of Toronto, Toronto, Ontario Canada; 4 Laboratory Medicine Program, University Health Network, Toronto, Ontario Canada; 5 Department of Computer Science, University of Toronto, Toronto, Ontario Canada; 6 Ontario Institute for Cancer Research, Toronto, Ontario Canada; 7 Department of Laboratory Medicine and Pathobiology, University of Toronto, Toronto, Canada; 8 Susan Leslie Clinic for Neuroendocrine Cancer, Sunnybrook Odette Cancer Center, Toronto, Ontario Canada; 9 Princess Margaret Cancer Centre, University Health Network, 101 College Street, TMDT, Room 9-305, Toronto, Ontario M5G 1L7 Canada

**Keywords:** Exome sequencing, Pancreatic neuroendocrine tumors, Molecular timing, Gene expression profiling, Whole-genome sequencing, Molecular cytogenetics, Loss of heterozygosity, Genetic instability, Publicly available data

## Abstract

**Background:**

Pancreatic neuroendocrine tumors (PANETs) are rare, slow growing cancers that often present with local and distant metastasis upon detection. PANETS contain distinct karyotypes, epigenetic dysregulation, and recurrent mutations in *MEN1*, *ATRX*, and *DAXX* (*MAD+*); however, the molecular basis of disease progression remains uncharacterized.

**Methods:**

We evaluated associations between aneuploidy and the MAD+ mutational state of 532 PANETs from 11 published genomic studies and 19 new cases using a combination of exome, targeted panel, shallow WGS, or RNA-seq. We mapped the molecular timing of MAD+ PANET progression using cellular fractions corrected for inferred tumor content.

**Results:**

In 287 PANETs with mutational data, MAD+ tumors always exhibited a highly recurrent signature of loss of heterozygosity (LOH) and copy-number alterations affecting 11 chromosomes, typically followed by genome doubling upon metastasis. These LOH chromosomes substantially overlap with those that undergo non-random mis-segregation due to ectopic CENP-A localization to flanking centromeric regions in *DAXX*-depleted cell lines. Using expression data from 122 PANETs, we found decreased gene expression in the regions immediately adjacent to the centromere in MAD+ PANETs. Using 43 PANETs from AACR GENIE, we inferred this signature to be preceded by mutations in *MEN1*, *ATRX*, and *DAXX*. We conducted a meta-analysis on 226 PANETs from 8 CGH studies to show an association of this signature with metastatic incidence. Our study shows that MAD+ tumors are a genetically diverse and aggressive subtype of PANETs that display extensive chromosomal loss after MAD+ mutation, which is followed by genome doubling.

**Conclusions:**

We propose an evolutionary model for a subset of aggressive PANETs that is initiated by mutation of *MEN1*, *ATRX*, and *DAXX*, resulting in defects in centromere cohesion from ectopic CENP-A deposition that leads to selective loss of chromosomes and the LOH phenotype seen in late-stage metastatic PANETs. These insights aid in disease risk stratification and nominate potential therapeutic vulnerabilities to treat this disease.

## Background

Pancreatic neuroendocrine tumors (PANETs) are rare neuroendocrine malignancies largely derived from pancreatic islet alpha- [1] and beta-cells [2]. Approximately half of all PANETs are non-functional, defined as the absence of hormone secretion, and thus resulting in asymptomatic progression and late detection that typically co-occurs with liver metastasis [1]. These tumors are characterized by mutations in chromatin modifiers *MEN1*, *ATRX*, and *DAXX* (MAD) (in 46, 18, and 31% of tumors, respectively) [3–7] and typically dichotomize into a genome that is either highly aneuploid or largely diploid with few copy-number variants (CNVs) [8–17]. Scarpa et al. defined a subtype of PANETs with a recurrent pattern of whole chromosomal loss (RPCL) in chromosomes 1, 2, 3, 6, 8, 10, 11, 15, 16, and 22 [17], while Stumpf et al. defined recurrent gains in the complementary set of chromosomes [8] suggesting a link via whole-genome duplication mechanisms or a technical difference in data normalization. The RPCL subtype is enriched for MAD mutations as well as an alternative lengthening of the telomere (ALT) phenotype [17] which indicates a potential functional link between the two. A recent characterization of PANETs highlights the role of epigenetic modifications into distinct subtype of this disease [2].

DAXX co-immunoprecipitates with both menin and ATRX via its C-terminal [18] and N-terminal regions, respectively [19]. The menin-DAXX complex assembles on DNA where DAXX is unoccupied by histone variant H3.3/H4 and functions to enhance marks of H3K9me3 at the promoter of membrane metallo-endopeptidase (MME), a colorectal cancer oncogene [20–22]. The DAXX-ATRX complex participates in a functionally distinct pathway, catalyzing replication-independent deposition of the histone variant H3.3 at telomeric and pericentric heterochromatin regions [23–25]. Directly associated with H3.3 is the H3 variant, CENP-A [26], a histone protein that is responsible for assembling kinetochore proteins and dependent upon *DAXX*- [18, 25, 27, 28] and *ATRX*-mediated [24, 29–32] histone modifications for its endogenous localization [33, 34]. Dysregulation of DAXX induces mis-localization of CENP-A, resulting in chromosomal instability, neocentromere formation, and micronuclei formation, a common result of premature sister chromatid separation [35].

In our study of 532 PANETs, we sought to understand the pathogenesis of PANETs by examining the relationship between MAD mutations, chromosomal instability, cohesion, and CENP-A localization. We found that MAD mutations (MAD+) in PANET tumors were strongly predictive of a highly conserved pattern of loss of heterozygosity (LOH) and copy-number (CN) alterations across select chromosomes, typically followed by genome doubling in late-stage disease or metastatic disease. These patterns of chromosome mis-segregation are likely to stem from mis-localization of CENP-A in *DAXX*-deficient cells, resulting in merotelic attachments and premature sister chromatid separation via cohesion fatigue. Herein, we show that an aggressive subtype of PANETs follows a conserved progression of molecular events that originates from non-random chromosome mis-segregation and may suggest potential therapeutic targets to disrupt this choreography.

## Methods

### Tissue acquisition

Our whole-exome sequencing (WES) cohort originated from 4 patients enrolled in the NET-SEQ study (ClinicalTrials.gov, NCT02586844) at the Princess Margaret Cancer Centre. Of the 7 patients registered in this study, 4 had histological or cytological diagnosis as well-differentiated pancreatic neuroendocrine tumors (PANETs) to be used for exploratory analysis. Our shallow whole-genome (sWGS) cohort was comprised of 15 NET samples provided by the Ontario Tumour Bank. Three sample types were processed: buffy coat blood cells, formalin-fixed paraffin-embedded (FFPE) tissues at time of diagnosis, and fresh-frozen core needle biopsies.

### Genomic characterization

We sequenced DNA from the WES cohort to target a depth of 250× coverage in tumors and 50× coverage in normals. We also generated RNA sequencing (RNA-seq) libraries from these cases, which we sequenced using ~ 80 million reads. We sequenced DNA from the sWGS cohort to 0.34× mean coverage. Sequence data were aligned to the human reference genome sequence build hg19. Variant detection in exome data was performed using MuTect [36] and HaplotypeCaller [37], while copy-number profiles were called using VarScan2 [38] and Sequenza [39]. Loss of heterozygosity data was inferred from both DNA and RNA data by determining purity-adjusted allelic fractions. Gene-wise transcript abundances were quantified using the Cufflinks suite of tools [40]. Pseudo allele-specific copy-number profiles were estimated from sWGS data using 500 kB bins tiled across the genome to count the number of reads and the number of heterozygous variants in each bin. To validate these copy-number calls, we paired this analysis with fluorescence in situ hybridization on complementary FFPE tissues.

### CENP gene expression analysis

We analyzed 148 PANET gene expression profiles obtained from published microarray datasets: 99 generated by Sadanandam et al. [41] (GSE73338) and 49 from Chan et al. [1] (GSE117851). We compared expression patterns to a set of normal pancreatic islet cells from 57 non-diabetic and 20 diabetic donors (GSE41762) generated by Tang et al. [42]. To approximate whether the CN signature was retained in MAD+ PANETs from these datasets, we first separated samples based on whether they carried MAD mutations and computed the *z*-score for gene expression against the MAD− PANETs on a per gene basis. Genes were mapped back to the human genome assembly hg19, and a loess regression with a 50% smoothing span was fitted to these values.

To calculate whether genes near the centromeres in LOH chromosomes are lower expressed than the rest of the chromosomal arm, we took the aforementioned gene expression *z*-scores and calculated the arm-level gene expression percentile and fractional distance to the centromere. Chromosomes were stratified into LOH and heterozygous chromosomes, and a loess regression was fit to the gene-level *z*-scores. To estimate regions of the chromosome arm that were repressed or elevated relative to the rest of the arm, we used an arm-level empirical cumulative density function to estimate the percentile of each gene.

### Detection of monoallelic expression

To detect monoallelic expression of genes, we called all SNPs from RNA-seq data using HaplotypeCaller [37]. We tested each gene containing 2 or more SNPs for MAE using a weighted *t* test. The allelic fractions of all SNPs in a gene, weighted by the number of reads supporting that SNP call, were compared to all SNP allelic fractions across the entire sample. By bootstrapping this calculation 1000 times per gene, we obtained the average *z*-statistic for each gene and compared it to a null distribution created using a similar test where the gene set is replaced with randomly selected SNPs.

### Detection of parental skewing

SNPs from WES data of NET-001 tumor, matching blood DNA, and maternal DNA were estimated using HaplotypeCaller. All SNPs were divided into groups based on chromosomes and were then discretized into either homozygous (AF ≥ 0.8 or AF ≤ 0.2) or heterozygous (AF > 0.2 and AF < 0.8). We only focused on SNPs that were homozygous in the maternal DNA and heterozygous in the NET-001 germline DNA. For each LOH chromosome, we calculated the fraction of SNPs that were homozygous and either matched the maternal SNPs or did not (paternal), or were heterozygous.

### CENP-A ChIP analysis

WIG files for the Nechemia dataset were downloaded from GEO:GSE111381 [43], while BigWIG files for the Nye dataset were downloaded from GEO:GSE120230 [35]. Peaks were assigned to cytobands based on the hg19 reference genome. CENP-A peaks were summarized across a reference “merged peaks” representation defined by Nye et al. using two metrics: the max peak height for each merged peak or reads per kilobase of peaks per million mapped reads (RPKM).

For the Nye dataset, overlapping peaks between *DAXX* and control groups were compared using a *t*-statistic. To test for an elevated number of peaks in each cytoband, we calculated the Kolmorogov-Smirnov *D*-statistic by comparing the peaks found only in that cytoband against peaks found across the entire genome.

### Alternative lengthening of telomere

Telomere lengths for all sWGS data were analyzed using Telomerecat [44]. Samples were split between PANETs and GINETs, and a one-sided *t* test was done on the estimated telomere lengths. A one-sided *F* test was also conducted to calculate for difference in variance.

### Meta-analysis of published datasets

Whole-exome sequencing of the BON-1 and QGP-1 PANET cell line from Vandamme and colleagues [45] was re-analyzed, and LOH segments were called based on allelic fractions (European Nucleotide Archive study ID: PRJEB8223). Copy-number profiles derived from CGH microarray data were obtained from data tables described in six publications [8–16] and transcribed into genomic coordinates (Additional file 1) by mapping to cytobands using the UCSC Table Browser hg19 cytoBandIdeo file (http://hgdownload.cse.ucsc.edu/goldenPath/hg19/database/cytoBandIdeo.txt.gz). Each copy ratio segment was assigned a value corresponding to the copy-status. Jaccard index values were calculated to measure the asymmetric binary concordance between any two copy-number profiles.

### Molecular timing in project GENIE

Copy-number profiles and mutational data of PANETs from AACR’s project GENIE (v1.0.1) were downloaded from Sage Synapse (https://www.synapse.org/; synapse IDs: syn7851250, syn7851253, and syn7851246). In total, 43 PANET samples had both copy-number information and mutational information. The molecular timing of these samples was determined by estimating the tumor purity required for every possible copy-number profile to generate the observed tumor purity for all somatic mutations. The simplest copy-number profile that fits the constraints of pathologist purity ± 0.15 and copy-number constraints imposed by the relative copy-states of somatic mutations was used to infer molecular timing of the disease.

Additional methods and detailed version and parameter information are available in the Additional file 2.

## Results

### Mutations in *MEN1*, *ATRX*, and *DAXX* are characteristic of chromosome mis-segregation errors

To characterize the molecular profiles of PANETs (Additional file 3: Fig. S1), we generated an exome and whole-genome dataset totaling 19 samples. The exome cohort consisted of whole-exome sequencing (WES) paired with RNA-seq across 4 patients, 6 samples (4 metastatic samples, 2 of which are complemented with their diagnostic tissue) (Additional file 4: Table S1a). Our whole-genome cohort consisted of 13 PANETs analyzed using shallow (0.3×) whole-genome sequencing (sWGS) and a 21-gene panel targeted deep sequencing, paired with FISH of 4 centromeric probes across 5 of the 13 samples (Additional file 4: Table S1b). Moreover, we included 165 PANET samples from version 5.0 of the publicly available AACR GENIE dataset [46] (Additional file 4: Table S1c).

**Fig. 1:**
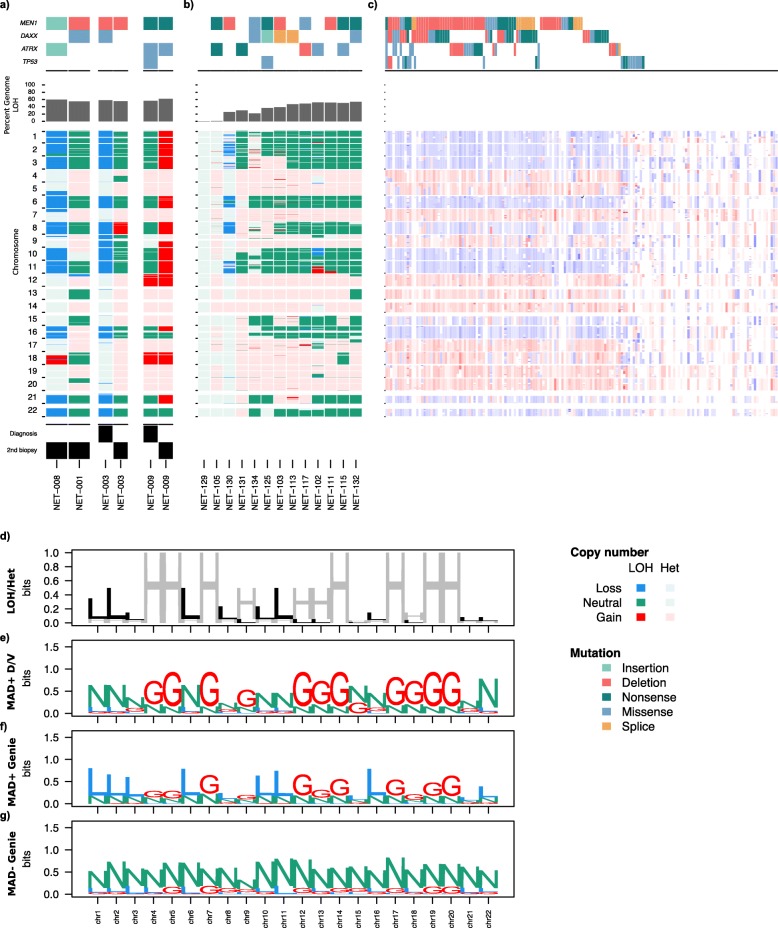
Loss of heterozygosity and copy-number profiles for PANET samples. Loss of heterozygosity profiles depicted as being copy-loss/haploid (blue), copy-neutral/diploid (purple), or copy-gain/triploid+ (red) for each PANET sample in the **a** exome, **b** whole-genome, and **c** AACR GENIE cohorts. Motif plots describe the most recurrent zygosity (**d**) or copy-number (**e**–**g**) states for each chromosome. MAD+ PANETs in the exome and whole-genome cohorts depict patterns of copy-neutral and copy-gain (**e**), while PANETs in the AACR GENIE cohort depict patterns of copy-loss and copy-gain for MAD+ samples (**f**) and copy-neutral for MAD− samples (**g**)

By stratifying our cohort based on MAD status (MAD+ *n* = 18/19), we discovered a highly recurrent copy-number and LOH pattern that overlapped the RPCL pattern described by Scarpa et al. [17] in our exome and whole-genome cohorts (Fig. 1a, b). Three cases in our whole-genome cohort did not exhibit this LOH pattern; this pattern is undetected in NET-105 due to low tumor purity obscuring the signal (purity = 0.25, Additional file 4: Table S1b), while NET-130 and NET-131 did not show LOH in chromosomes 15, 16, 21, and 22. Although the inferred ploidy between samples differed, almost every case demonstrated LOH for chromosomes 1, 2, 3, 6, 8, 10, 11, 16, 21, and 22 and retained heterozygosity for chromosomes 4, 5, 7, 9, 12, 13, 14, 17, 19, and 20 (Fig 1d). Chromosomes 15 and 18 showed no consistent pattern of variation with heterozygosity and LOH occurring in equal proportions. Moreover, the LOH chromosomes were largely copy-neutral (i.e., diploid) while the heterozygous chromosomes showed copy-gain (Fig. 1e), mimicking the pattern observed by Stumpf et al. [8–10]. We validated the CN and LOH regions identified in our WES samples using the Affymetrix SNP 6.0 array (Additional file 3: Fig. S2a) and allelic skewing in RNA sequencing (Additional file 3: Fig. S2b). Additionally, we validated the CN inference in our 13 sWGS samples using fluorescent in situ hybridization of centromeric probes targeting LOH chromosomes 3 and 10, and zygosity-intact 7 and 17 (Additional file 4: Table S2a). To rule out the possibility of germline LOH, we confirmed that all patients had a diploid heterozygous genome in their germline DNA (Additional file 3: Fig. S3a).

### Extended validation of CN and LOH signature

Next, we sought to expand our validation through inclusion of a larger, clinically derived cohort made available through the AACR GENIE consortium [46]. While genome-wide zygosity calls were not available for the GENIE cohort, we were able to stratify the CN profiles of 165 PANETs samples into MAD+ (*n* = 99) and MAD− (*n* = 66) subgroups. Consistent with our genome-wide cohort, the targeted clinical panel sequencing data recapitulated the same pattern of losses and gains (Fig. 1c) and a near perfect overlap of MAD+ karyotypes. Chromosomes 8, 9, 15, and 21 had more than one prominent copy-states, suggesting more variable copy-number alterations of these chromosomes (Fig. 1f). PANETs in the GENIE cohort without MAD mutations were largely diploid with fewer recurrent gains and losses compared to those with MAD mutations (Fig. 1g).

We defined a CN signature by utilizing all available CN aberrations (CNA) data to calculate the co-occurence of copy-number states between chromosomes. By taking the copy-number states with the highest propensity (Fig. 1f), we flagged aberrations that are synchronous in their presentation from those that are random independent events (Additional file 3: Fig. S4a). Furthermore, we identified loss of chromosome 7 (Additional file 3: Fig. S4b) and gain of chromosomes 1, 6, and 16 (Additional file 3: Fig. S4d) as aberrations strongly antagonistic of our copy-number signature. Since the aberrations are largely chromosomal in size, we hypothesized that these CNA were likely a result of mis-segregation errors from merotelic events resulting in lagging chromosomes.

To evaluate whether PANET model systems accurately recapitulate these well-defined molecular signatures, we inferred genome-wide zygosity using publicly available WES data from two metastatic PANET cancer cell lines, BON-1 and QGP-1 [45]. While both cell lines exhibited a high degree of aneuploidy and LOH, neither were MAD+ and the affected chromosomes differed dramatically between cell lines and when compared to the MAD+ PANETs in our meta-analysis (Additional file 3: Fig. S5). These results are in agreement with those of Boora et al. [47], suggesting that BON-1 and QGP-1 are genetically distinct from clinical samples of MAD− and MAD+ PANETs and should be used with caution in understanding PANET cancer biology or for pharmacological screening.

### The MAD phenotype is associated with alternative lengthening of telomeres

To test whether MAD+ PANETs in our cohort exhibited ALT phenotype as reported by Jiao et al. [45, 48], we compared the overall length of telomeres between 13 PANETs and 10 MAD− GINETs (gastrointestinal neuroendocrine tumors) using sWGS. We observed longer telomere lengths in PANETs (*p* = 0.031; one-sided *t* test) as well as greater variation across samples (*p* < 0.001; one-sided *F* test) suggestive of an ALT phenotype (Additional file 3: Fig. S6). In the sWGS cohort, NET-129 lacked any MAD mutations and displayed telomere length concordant with the average length of GINET telomeres. The presence of ALT might suggest disruption of H3.3 incorporation, which we hypothesize is linked to the chromosomal mis-segregation pattern observed.

### Mis-segregation errors are associated with *DAXX*-linked cohesion fatigue

The MAD+ CN signature may be a result of selective pressures favoring the retention and loss of certain chromosomes, or merely a stochastic event that always leads to the same karyotype. We first hypothesized that retention of chromosomes may be a consequence of selective pressures due to monoallelic expression (MAE). By examining 36 PANETs (23 MAD+, 13 MAD−) from our study and Chan et al. [1] that exhibited the LOH signature (Additional file 3: Fig. S7), we only found 11 genes that exhibited evidence of MAE scattered across 7 of the 8 chromosomes that were always heterozygous, leaving chromosome 14 unexplained (Additional file 3: Fig. S8a). Our second hypothesis was that there is a genetic predisposition to losing chromosomes from one parent. For NET-001, we flagged heterozygous single-nucleotide polymorphisms (SNPs) in germline DNA that were observed to be homozygous in the matched tumor and germline DNA collected from the patient’s mother. We determined that only 8 of the 11 chromosomes exhibiting LOH were of maternal origin (Chr1, 6, 8, 11, 13, 15, 21, and 22) and 3 were paternal (Chr2, 3, and 18) (Additional file 3: Fig. S8b), hence showing no statistical significance for favoring parental origin (*p* = 0.23, binomial test). Overall, we show that there are no robust selective factors driving our copy-number signature suggesting that another mechanism may be at play.

**Fig. 2:**
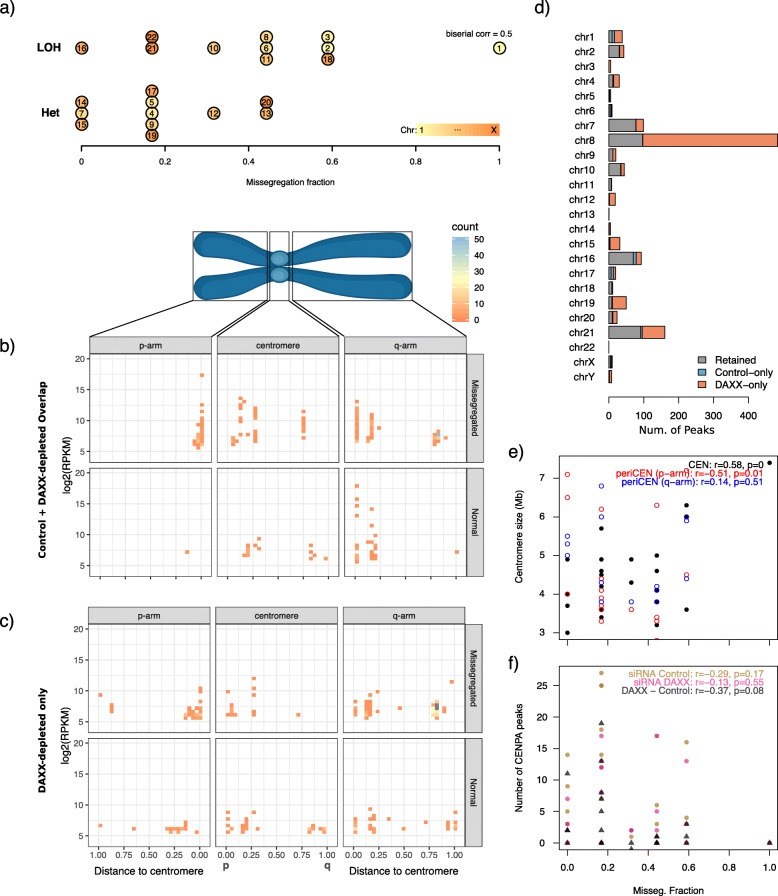
Ectopic CENP-A loading localizations in DAXX-depleted and wild-type SW403 colorectal cancer cell lines from the Nye et al. dataset. **a** Mis-segregated chromosomes identified by Worrall et al. through single-cell analysis, compared to the LOH chromosomes we define. **b**, **c** Chromosome-relative localization of CENP-A peaks that are found in either DAXX-depleted-only regions (**b**) or peaks that are found in both DAXX-depleted and control cells (**c**) for both the commonly mis-segregated and normal-segregation chromosomes as identified by Worrall et al. **d** Depiction of acquired, maintained, and lost CENP-A peaks when SW403 undergo DAXX depletion. **e** Correlation plots between mis-segregation fractions and size of the centromere (CEN), or the cytobands immediately flanking the centromere (periCEN) on the p-arm or q-arm. **f** Correlation plots between the number of CENP-A peaks and mis-segregation fractions

A recent publication by Worrall et al. [49] details a non-random chromosome mis-segregation pattern in SW403 colorectal cancer cells similar to our own (Fig. 2a; *r* = 0.50, point-biserial correlation) that is due to cohesion fatigue, resulting in premature sister chromatid separation and lagging chromosome formation. To explore the possibility of centromere dysfunction as the underlying mechanism driving formation of the MAD+ CN signature, we integrated our genomic data with two ChIP-seq datasets characterizing CENP-A binding locations. Nechemia-Arbely et al. defined centromeric CENP-A loads in HeLa cells throughout the cell cycle under endogenous and ectopic CENP-A expression [43] (Additional file 3: Fig. S9). Nye et al. characterized non-centromeric CENP-A binding in DAXX-depleted and DAXX-intact SW480 colorectal cancer cells [35] (Fig. 2b, c).

In HeLa cells, chromosomal CENP-A levels in the centromere were more similar between endogenous and ectopic expression conditions than between cell cycle phase, with chromosomes 2 and 9 containing the highest level of deposition and chromosomes 14, 19, and 21 the lowest (Additional file 3: Fig. S9). Meanwhile, in SW480 cells, there was an increase of ectopic CENP-A occupancy from 397 to 1124 kb when DAXX was depleted, the majority of this coverage occurring in chromosomes 8, 19, and 21 while losing coverage on chromosomes 1, 16, and 17 (Fig. 2d).

Using the single-cell sequencing (SCS) data from Worrall et al., we tested whether mis-segregation of specific chromosomes were related to centromeric CENP-A level and centromere size [49]. We did not observe any correlation between CENP-A levels in centromeric regions and mis-segregation rates (*r*_Endogenous_g1,g2_ = − 0.005, − 0.09, *r*_Elevated_g1,g2,RC_ = − 0.11, 0.01, − 0.41), nor with the coverage or number of CENP-A binding sites acquired in DAXX-depleted conditions (*r*_coverage_ = 0.08, *r*_count_ = 0.12). However, there was a significant correlation between the frequency of mis-segregation per chromosome and the size of centromeres as well as flanking cytobands (hg19: *r*_CEN_ = 0.58, *p* < 0.01; *r*_flank_ = 0.51, *p* = 0.01; Fig. 2e). The features that most correlated with chromosomal mis-segregation rates were the mean CENP-A levels across all ectopic locations (*r*_DAXX_ = 0.51, *p* = 0.02; *r*_Control_ = 0.43, *p* = 0.06) and levels in regions close to the centromeric regions under both DAXX-depleted and control conditions (*r*_DAXX_ = 0.56, *p* = 0.02; *r*_Control_ = 0.58, *p* = 0.02; Fig. 2f). While the majority of ectopic CENP-A peaks localized to the flanking regions of centromere (Fig. 2b), we found that newly acquired CENP-A peaks in DAXX-depletion conditions were primarily localized to these flanking regions (Fig. 2c). These results suggest that DAXX-deficient tumors may mis-localize CENP-A to ectopic sites that flank the centromere, which could possibly seed the formation of neocentromeres and favor merotelic attachments of select chromosomes.

### LOH chromosomes in MAD+ PANETs exhibit a gene-repressive environment directly adjacent to the centromere

**Fig. 3:**
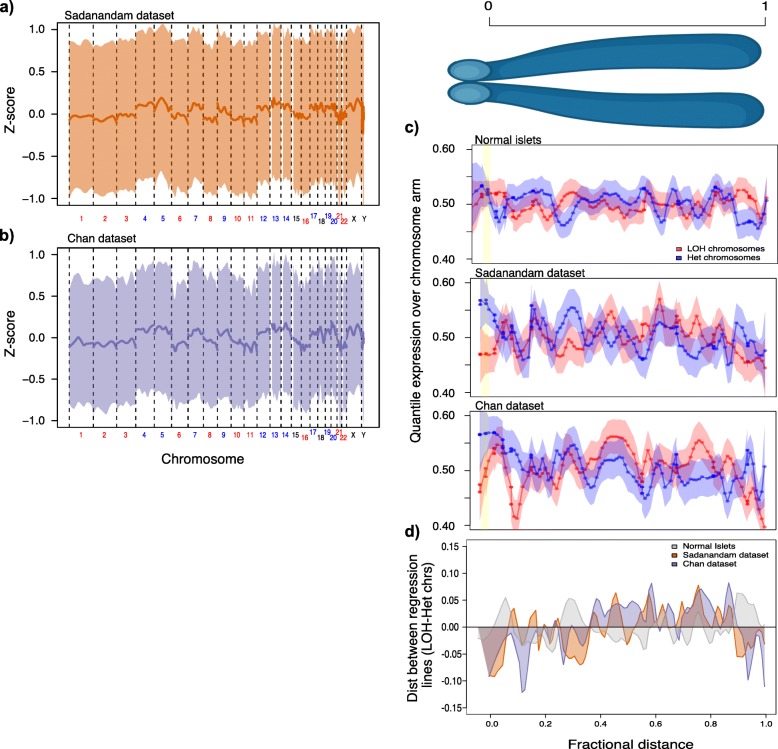
Gene expression recapitulating the copy-number signature of MAD+ PanNETs. **a**, **b** The copy-number signature was inferred from RNA-seq data from the Sadanandam (*n*_MAD+_ = 28, *n*_MAD−_ = 47) and Chan (*n*_MAD+_ = 30, *n*_MAD−_ = 17) datasets by calculating the *z*-score on a per-gene basis using MAD+ PANETs compared to MAD−. These plots visualize data from the **a** Sadanandam dataset and the **b** Chan dataset. **c**, **d** Regions of a chromosome arm that have elevated or repressed gene expression in MAD+ samples for the LOH chromosomes (red) relative to genes on heterozygous chromosomes (blue) are plotted against the fractional distance to the centromere (0 = at centromere boundary, 1 = chromosomal arm end). Three datasets are illustrated here: the Tang et al. dataset composed of 77 normal pancreatic islets (top), the Sadanandam PANET dataset (middle), and the Chan PANET dataset (bottom). **d** Distances between loess regression lines of LOH to heterozygous chromosomes where all 3 aforementioned datasets are overlapped on each other to better visualize overlapping and discordant regions relative to normal islet cells

It has been shown that proximity to chromocenters and pericentromeric regions results in gene repression [50]. Therefore, to test whether PANETs have increased CENP-A loading in a similar fashion to DAXX-depleted colorectal cells, we tested whether there is a corresponding decrease of gene expression in regions proximal to the centromere in the LOH chromosomes. We examined the gene expression profiles of 122 PANETs with known MAD mutational status from two datasets: Sadanandam et al. [41] (28 MAD+, 47 MAD−) and Chan et al. [1] (30 MAD+, 17 MAD−). We first verified that the MAD+ samples recapitulate our previously defined copy-number signature (Fig. 3a, b). By normalizing the expression scores of each gene for all genes on the chromosome arms, we sought to identify whether there were regions on the chromosome that had lower or higher expression. We observed that there was a region, between 0 and 0.025 fractional distance of the centromere to the end of the chromosome arms, which had decreased gene expression in LOH chromosomes relative to heterozygous chromosomes (Fig. 3c, d). While this observation was noted in both datasets, it was noticeably absent in a dataset composed of 77 normal pancreatic islet cells [42] (Fig. 3c). The minor discrepancies between PANET datasets may reflect the inherent noise in RNA-seq data, the stratification of LOH and heterozygous chromosomes without genome data to validate, or the simplifying assumption that chromosomal arms only have single copy-state. As seen in the colorectal cell lines, the increased deposition of CENP-A in the pericentromeric region due to DAXX depletion may be linked to a corresponding decrease of gene expression in this region, suggesting an unseen mechanism for chromosomal mis-segregation pattern in PANETs.

### Mutational events in MAD genes precede chromosomal mis-segregation

Given the progression of events proposed by our mis-segregation model, we assessed whether MAD mutations arose prior to LOH events. Hence, we developed a molecular timing analysis for the initial release of AACR GENIE dataset (Additional file 3: Fig. S1) [46]. We obtained allelic fractions for clinical panel sequencing data from 43 mixed primary/metastasis samples (29 MAD+, 14 MAD−) that contain both copy-number and somatic mutation data in the GENIE v1.0 data freeze. Of the MAD+ population, 26/29 samples co-occur with the CN signature versus only 1/14 MAD− samples (Additional file 3: Fig. S10).

**Fig. 4:**
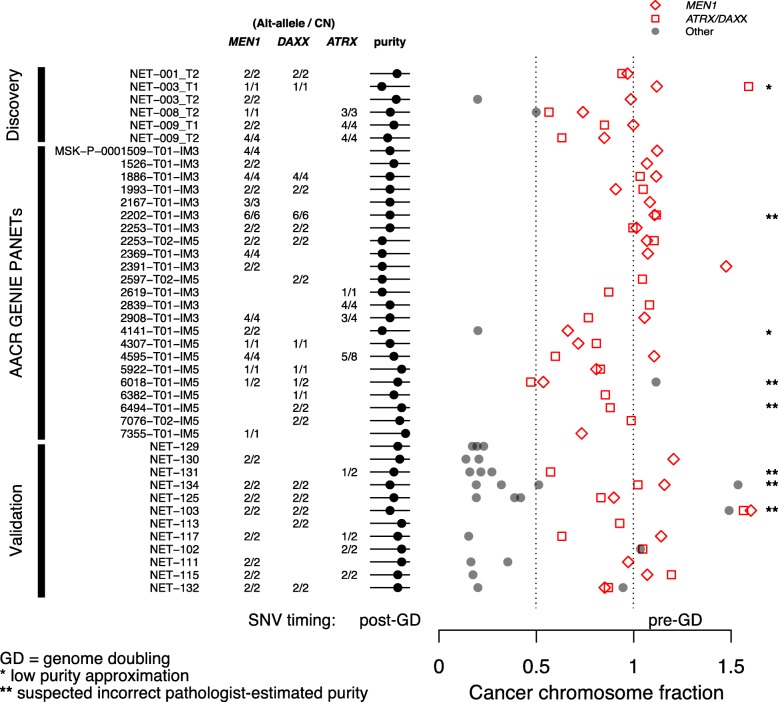
Cancer chromosome fraction for MAD genes in the GENIE PANET samples. Estimations of the theoretical tumor allelic fraction for *MEN1* (red diamond), *DAXX/ATRX* (red square), and other gene-level mutations (gray circles) for the copy-number model (number of ALT alleles/ploidy) that best represents the pathologist-estimated purities across the different cohorts. A fraction of 1.0 indicates a homozygous variant, and 0.5 a heterozygous variant. Any deviations from these values represent variance in the observed allelic fractions

We next estimated the allele-specific CN profile of the GENIE cohort using the observed allelic fractions, CN log2 ratios, and pathologist-estimated tumor purities (± 0.15) (Supplementary Data). Of the MAD+ GENIE PANETs, 6/29 samples with low (< 30%) tumor cellularity were excluded from the analysis. The remaining 23 PANETs showed a strong tendency to adopt a CN model with cancer cell fraction of MAD mutations at 1.0, reinforcing the hypothesis that these mutations occur prior to LOH and genome doubling events (Fig. 4). We observed a significant enrichment of *MEN1* and *DAXX* mutations prior to LOH and genome doubling events (Bonferroni adjusted *p* values: *MEN1* = 0.00029, *DAXX* = 0.00011, binomial test) when using a cutoff of 0.85 cancer cell fraction. *ATRX* mutations reached significant enrichment at a cancer cell fraction cutoff of 0.63, which is expected as LOH on the X chromosome occurs infrequently in PANETs and may not always require LOH as a second hit due to X-inactivation. We observed that 35/39 MAD+ PANET samples follow a molecular timing model of MAD mutations prior to LOH (4/4 exome cohort, 10/12 whole-genome cohort, 21/23 GENIE cohort) (*p* = 3.4 × 10^−7^, binomial test). Collectively, our results provide evidence that acquisition of *MEN1* and *DAXX/ATRX* mutations is an early event that leads to a genome-wide LOH event, likely through centromere fatigue from merotelic attachments.

### Meta-analysis of copy-number profiles informs the molecular progression towards late-stage PANETs

PANET CN profiles have been extensively reported in the literature, each with their own reported chromosomes of significance. Scarpa et al. [17] examined 102 clinically sporadic PANETS and identified 4 CN groups: (G1) CN loss affecting > 50% of the genome, (G2) a diploid genome with little to no LOH or CN loss, (G3) CN gains affecting ~ 100% of the genome, and (G4) a mix of CN-LOH and CN gains. The absolute copy-number profiles of our exome cohort revealed 3 of the 4 groups described by Scarpa et al. The pancreatic diagnostic sample for patient NET-003 displays the G1 signature while the liver-metastasis sample displayed the G4 CN-LOH signature. Similarly, NET-009 presented with a similar transition of the G4 CN-LOH signature towards a G3 whole-genome gain (Fig. 1a), suggesting a mechanism of whole-genome duplications underlying PANET progression.

To further validate these groupings across independent cohorts, we analyzed previously published CGH datasets for the same signatures of absolute loss of LOH chromosomes (G1) or gain of retained chromosomes (G4). Due to the inability of CGH to detect CN-LOH and whole-genome gains, we anticipated tumors with the G3 profile to appear similar to G4 profiles defined by no aberrations in the LOH chromosomes with gains of the retained chromosomes. To compare the CGH copy-number data with our current study, we performed a meta-analysis of 226 NETs from 8 previous reports (Supplementary Data) [8–16]. By clustering the absolute copy-number profiles of our NETs and published datasets (Online methods), we demonstrated that tumors were divided into 5 clusters characterized by high and low fractions of genome-wide aneuploidy (Additional file 3: Fig. S11a). PANETs in our exome and whole-genome cohorts were mostly represented in cluster 1 which best represented G4 PANETs. Cluster 5 displayed loss of LOH chromosomes, suggesting that they best represent G1 PANETs. Cluster 4 was composed of 9 samples but contained karyotype that is reminiscent of profiles from the GENIE cohort, suggesting that this may reflect a normalization error rather than true biology. The remaining clusters 3 and 5 were largely diploid with few recurrent CN aberrations such as chromosome 11 loss.

**Fig. 5:**
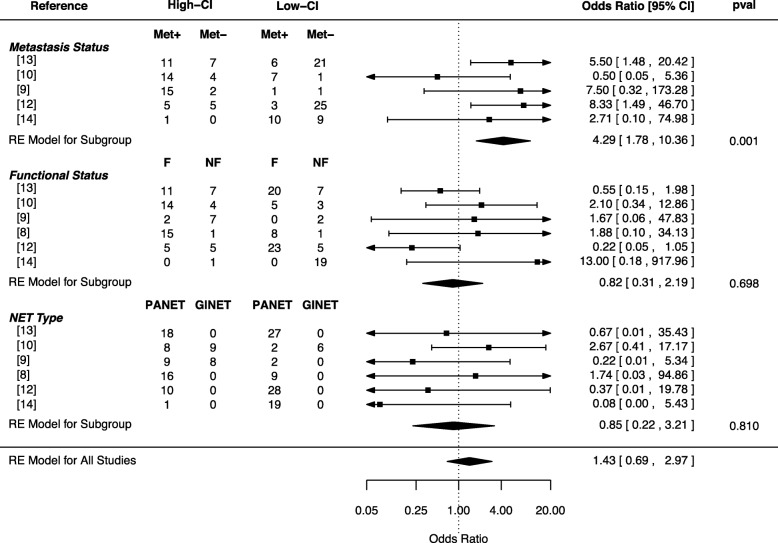
Meta-analysis of the CGH datasets for the highly aneuploid PANET tumors (High-CI) against the low aneuploid PANET tumors (Low-CI). The parameters being compared are the metastasis status of the tumor type (Met+, metastasis present; Met−, no metastasis) and the functional status (F, functional; NF, non-functional)

**Fig. 6:**
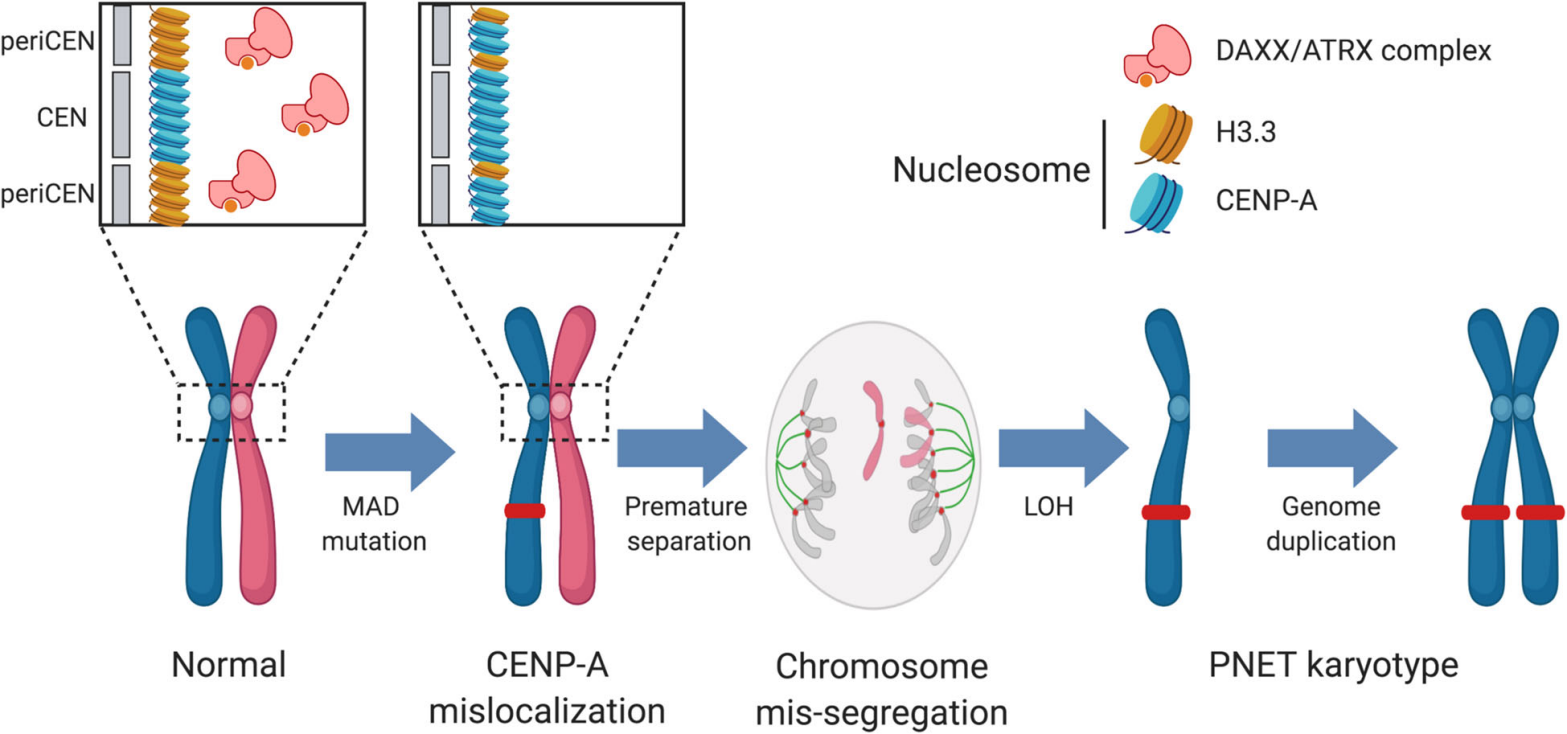
Proposed molecular progression mechanism for pancreatic neuroendocrine tumors. Normal islet cells acquire a mutation in *MEN1*, and *ATRX* or *DAXX* which leads to perturbed deposition of H3 histone variants H3.3 and CENP-A at nucleosomes in centromeric sites. This results in premature sister chromatid separation and loss of one allele, followed by a series of genome duplications

As PANETs with increased chromosomal instability are characteristic of late-stage and more aggressive PANETs [48], we next sought to validate whether PANETs with the CN signature were in fact more aggressive. We separated samples based on copy-number profiles with high-chromosomal instability (high-CI) or low-chromosomal instability (low-CI) (Additional file 3: Fig. S11b). PANETs with high-CI were more likely to be metastatic (OR 4.35, 95% CI [1.99, 9.52]; *p* = 0.00; Cochrane’s *Q*) (Fig. 5), and the majority of the high-CI NETs were those found in clusters 1, 4, and 5 (Additional file 3: Fig. S11a); all tumors follow the proposed model of PANET CN progression. Thus, loss of the LOH chromosomes appears to be an initial step towards metastasis, reflecting a transient state due to the small sample size, resulting in a more stable and metastatic genome-doubled PANET (Fig. 6).

## Discussion

In our study, we integrate large publicly available datasets of PANETs to show a remarkably conserved *MEN1*- and *DAXX/ATRX*-driven metastatic disease progression. Across 306 PANETs with inferable copy-number profiles and MAD mutational status, we observed a well-defined pattern of LOH affecting select chromosomes following somatic mutations of *MEN1* and *DAXX* or *ATRX*. By leveraging expression profiles for 122 of the 306 PANETs [1, 41], we show that this CN signature may be linked to perturbation of core kinetochore processes which would induce chromosomal mis-segregations. A recent publication from Worrall et al. suggests that there may be order in the timing of chromosomal mis-segregation stemming from merotelic attachments and cohesion fatigue [49]. Strikingly, their mis-segregation fractions from SCS partially overlapped our LOH signature, suggesting a potential mechanism that we investigated using large CENP-A ChIP-seq datasets [35, 43]. Our results support the hypothesis that depletion of DAXX is associated with increased chromosome-specific ectopic CENP-A deposition, resulting in decreased gene expression [50], that correlates with mis-segregation frequencies. Finally, using the AACR GENIE dataset [46], we show that *MEN1* and *DAXX/ATRX* mutational events all preceded the onset of chromosomal instability in clinical samples, subsequently resulting in LOH and whole-genome duplication to propagate chromosomal stability and increase tumor aggressiveness in 226 PANETs [8–16]. Overall, we define the molecular progression mechanisms for an aggressive subtype of PANETs which is also the first known observations to support the non-random chromosome mis-segregation theory [49] in primary clinical tumor specimens.

We observed that non-random mis-segregation of chromosomes in the SW480 colorectal cancer cell line [49] largely overlaps the LOH chromosomes in PANETs. This chromosome-specific overlap suggests that merotelic attachment and lagging chromosome formation is the common underlying mechanism guiding patterns of mis-segregation. Unequal chromosome and centromere sizes as well as levels of CENP-A can predispose chromosomes to merotelic attachment [42]*.* The “placeholder theory” posits that H3.3 acts as a placeholder at centromeric domains during S phase, to be replaced by CENP-A during late G1 [25]. H3.3 deposition at tandem repeat sites in centromeric and pericentromeric regions [27] is largely guided by the DAXX-ATRX complex. Disruption of H3.3 deposition is evident due to the presence of the alternative lengthening of telomere phenotype exclusively seen in the MAD+ PANETs [4, 24, 51, 52]. Using a dataset produced by Nye et al. that illustrates mis-localization of ectopic CENP-A in SW480 cells under DAXX-depleted conditions [35], we calculated a significant correlation with increased CENP-A deposition in regions flanking the centromere and mis-segregation fraction per chromosome. To translate these findings to PANETs, we observed a region immediately adjacent to the centromeres in LOH chromosomes in two published PANET datasets that had decreased gene expression relative to the rest of the chromosome arm. Thus, we propose that promiscuous CENP-A deposition to flanking centromeric regions is a factor that induces merotelic attachments, lagging chromosome formation, and the mis-segregation pattern proposed by Worrall et al.

The functional effects of aneuploidy are generally detrimental to cellular proliferation [53, 54] and can induce aneuploidy-associated stresses [55]. Loss of an entire chromosome can have drastic effects, resulting in slowing of cell growth [53] but may allow advantages through loss of tumor suppressor genes (TSG) [56]. Additionally, duplication of the remaining chromosomal region following a CN loss could harbor advantageous alterations, allowing cells to overcome the negative growth effects of chromosomal loss [57] or enhance for homozygous expression of preceding oncogenic mutations [58]. A study by Taylor et al. illustrates this point in lung epithelial cells where they used CRISPR-Cas9 to induce loss of chromosome 3p. After several passages of slow growth, the cells acquired whole-chromosome duplication to overcome the negative growth effects incurred from the aberration [57]. PANETs are well characterized as slow growing neoplasms that are clinically detectable only when they have metastasized [59]; the initial steps of disease progression described as near-global LOH could be an underlying mechanism for this slow growth. Acquired whole-genome duplication would be the tumor’s way to alleviate the negative growth effects of MAD-induced LOH, resulting in a more aggressive tumor that harbors loss of tumor suppressor genes but is not confounded by aneuploidy-associated stresses.

While the sample size of our in-house PANET cohort is small, we were able to leverage publicly available datasets allowing us to create a unifying model of disease progression to explain the remarkable consistency between karyotypes. However, due to the nature of this meta-analysis, we were unable to confidently validate our epigenetic dysregulation hypothesis due to the absence of publicly available ChIP-seq of H3.3 and CENP-A data in MAD+ and MAD− PANETs. Instead, we provide preliminary results leveraging work in SW480 and HeLa cells paired with evidence of a repressive gene environment proximal to centromeres in PANETs to provide compelling evidence to pursue further in vivo validation of this disease progression. Furthermore, we acknowledge that there are minor variations in copy-number and LOH profiles in the literature [16, 60, 61], but we hypothesize that these differences are a reflection of stochastic chromosomal instability events, mutational profiles allowing for more aneuploidy tolerance (e.g., *TP53* mutation), or synthetic lethality which may alter which chromosomal losses are tolerated.

## Conclusions

In our study, we observed a conserved trend of MEN1-, ATRX-, and DAXX-induced chromosome mis-segregation, leading to the characteristic karyotype of aggressive PANETs. These findings pave the way for functional validation studies to recreate the molecular progression of PANETs in model systems. Understanding the molecular basis of disease progression towards a more metastatic state has several benefits, specifically for risk stratification, treatment design for intermediate progression stages, and possibly even prophylactic treatment in at-risk individuals. As loss of heterozygosity appears as the molecular trigger for metastases, genome duplication acts as a mechanism to stabilize the genome.

## Supplementary information

**Additional file 1.** Details of CGH datasets, listing chromosome gains and losses as reported in the paper of origin.

**Additional file 2.** Supplementary methods for the manuscript.

**Additional file 3.** Supplementary figures for the manuscript.

**Additional file 4.** Supplementary tables for the manuscript.

**Additional file 5.** Somatic mutation calls from the exome and whole-genome cohort analyzed in this study.

**Additional file 6.** Cellular fraction estimates of SNVs from 43 samples from the AACR GENIE v1.0 cohort as well as the exome and whole-genome datasets generated in this study.

## Data Availability

All somatic mutations and copy-number aberrations from the exome and whole-genome cohort can be found in Additional files 5 and 6, while raw sequencing data is controlled access at EGAS00001004239 (https://www.ebi.ac.uk/ega/studies/EGAS00001004239). All CGH analyzed during this study are included in Additional file 1 as well as in the supplementary files of the original publications [8–16]. Publicly available gene expression profiles from PANETs were obtained from GEO datasets GSE73338 (doi: 10.1158/2159-8290.CD-15-0068) [41] and GSE117851 (doi: 10.1038/s41467-018-06498-2) [1]. Gene expression profiles from normal pancreatic islet cells were obtained from the GEO dataset GSE41762 (doi: 10.1126/scitranslmed.3009934) [42]. Data for CENP-A ChIP-seq data were obtained from GEO datasets GSE111381 (doi: 10.1101/428557) [43] and GSE120230 (doi: 10.1371/journal.pone.0205948) [35]. Whole-exome sequencing data from PANET cell lines were obtained from European Nucleotide Archive study ID: PRJEB8223 (doi: 10.1530/JME-14-0304) [45]. The datasets generated during and/or analyzed during the current study are available in the AACR GENIE repository, http://www.cbioportal.org/genie/ (doi: 10.1158/2159-8290.CD-17-0151) [46].

## Abbreviations

AACR: American Association for Cancer Research
ALT: Alternative lengthening telomere
CKG: Core kinetochore genes
CN: Copy number
CN-LOH: Copy-neutral loss of heterozygosity
CNA: Copy-number alterations
FFPE: Formalin-fixed paraffin-embedded
FISH: Fluorescence in situ hybridization
GENIE: Genomics Evidence Neoplasia Information Exchange
GINET: Gastrointestinal neuroendocrine tumor
GTEx: Genotype-Tissue Expression project
LOH: Loss of heterozygosity
MAD+: *Mutant MEN1* and *ATRX/DAXX*
MAD−: *Wild-type MEN1* and *ATRX/DAXX*
MAE: *Monoallelic expression*
NET: Neuroendocrine tumor
PANET: Pancreatic neuroendocrine tumor
RPCL: Recurrent pattern of whole chromosomal loss
RPKM: Reads per kilobase of peaks per million mapped reads
SCS: Single-cell sequencing
sWGS: Shallow whole-genome sequencing
TCN: Total copy number
TSG: tumor suppressor gene
WES: Whole-exome sequencing
