# Supplementary material for: Centromeric cohesion failure invokes a conserved choreography of chromosomal mis-segregations in pancreatic neuroendocrine tumor
Source: Genome Med. 2020 Apr 28;12:38. doi: 10.1186/s13073-020-00730-9 (PMC7189550; doi:10.1186/s13073-020-00730-9)
Supplement: Supplementary file 2 — Additional file 2. Supplementary methods for the manuscript. [file 13073_2020_730_MOESM2_ESM.pdf]

Supplementary Methods

All code and relevant data files can be accessed in the git repository:
https://github.com/pughlab/net-seq

Tissue acquisition

Our discovery cohort consisted of fresh-frozen core-needle biopsies of metastasized neuroendocrine tumours and matching blood samples originating from 7 patients enrolled in the NET-SEQ study at the Princess Margaret Cancer Centre. Eligible patient had histological or cytological diagnosis of well-differentiated neuroendocrine tumours (NETs) or well-differentiated pancreatic neuroendocrine tumours (PANETs); at least one biopsiable lesion deemed medically accessible and safe to biopsy; fulfill local institution's laboratory parameters for tumour biopsy, and provided written voluntary informed consent. Archived tumour samples, when available, were collected from all patients to perform additional genomic testing. For the validation cohort, NET samples from 38 patients were provided by the Ontario Tumour Bank, which is supported by the Ontario Institute for Cancer Research through funding provided by the Government of Ontario. A mix of fresh-frozen and formalin-fixed paraffin embedded (FFPE) tissues as well as matched blood buffy coat cells were collected based on availability. Of these tumours, 19 originated from patients diagnosed to have a primary PANET (9/19 are metastatic) and 19 with a primary GINET (16/19 are metastatic). All tissues were reviewed by a reference pathologist to confirm diagnosis of NET.

Sample preparation

Three sample types were processed: buffy coat blood cells, FFPE tumours, and fresh-frozen tumours. Fresh-frozen tissues were embedded in optimal cutting temperature (OCT) compound and blocks subsequently sectioned to collect 10x10µm curls. Slides were prepared by sectioning tumours 6-10µm and mounting them to positively charged glass microscope slides. Slides were fixed in 95% ethanol and staining protocol according to Pena-Llopis & Brugraolas, 2013 [1] (p.26) with the following modifications: step 1-2,4,9-10 excluded, step 14 2x emerged in xylene. High differentiation was performed in step 7 using 1.0% HCl in 70% EtOH solution. Extraction of DNA from buffy coat blood cells was done using the Qiagen Gentra-Puregene kit. DNA and RNA were co-isolated from formalin-fixed paraffin embedded tissues using AllPrep DNA/RNA FFPE Kit and the QIAamp DNA micro kit. Co-isolation of fresh-frozen tissues were processed using AllPrep DNA/RNA Mini Kit for DNA isolation and mirVana™ miRNA Isolation Kit with phenol (Ambion) for total RNA isolation or the Qiagen AllPrep-DNA/RNA/miRNA Universal Kit.

Exome and RNA sequencing

For exome sequence analysis, we performed hybrid selection of 200 ng of genomic DNA to isolate 75 Mb of genomic DNA representing coding exons and untranslated regions of genes annotated by the Consensus Coding Sequence, RefSeq, GENCODE, miRBase, and UCSC Gene databases (Agilent SureSelectXT Human All Exon V5+UTRs kit). Through the infrastructure of the Princess Margaret Genomics Centre (PMGC, www.pmgenomics.ca), captured fragments were sequenced to greater than 250X median coverage using 100 bp paired-end reads on Illumina HiSeq 2000 Genetic Analyzers. To detect somatic alterations, we compared WES data from each tumour with a matched lower coverage (50X median) exome sequence derived from the patient's peripheral leukocytes. Raw reads were aligned to the hg19 reference genome using the BWA 0.7.9a aligner [2]. For RNA-seq, we used the Illumina TruSeq Stranded Total RNA kit applied to 200 ng of total RNA. For each tumour, we generated >80 million pair-end 100 bp reads. The raw reads were aligned to the hg19 reference genome using STAR v2.4.2a RNA-seq aligner [3]. For both DNA and RNA data, aligned reads were then processed using the Mark Duplicates, Indel Realigner and Base Recalibration modules of the GATK v3.2.2 suite according to GATK Best Practices workflow [4–6]. All downstream analysis was based on these co-cleaned alignment mappings.

Targeted sequencing

A 21-gene panel for pancreatic neuroendocrine tumours was designed using xGen Predesigned Gene Capture Pools: MEN1, DAXX, ATRX, PTEN, TP53, CDC42BPB, DST, DEPDC5, KLF7, BCOR, PRRC2A, URGCP, ARID1A, ZNF292, DIS3L2, KMT2C, SETD2, MUTYH, BRCA1, BRCA2, and CHEK2. A total of 1,439 probes were used, covering 140,184 bases for a total of 99.09% coverage of the targeted genes.

Variant calling

To map regions of LOH, variant calling was done using GATK HaplotypeCaller [6] for both WES and RNA-seq data. The allelic fraction for each identified variant was plotted according to their respective genomic coordinates. Circular binary segmentation (CBS), from the "DNAcopy" v1.44.0 R package [7], was then used to identify stretches of allele fractions that corresponded to either the reference or alternate allele. The threshold for loss-of-heterozygosity regions were set by computationally estimated tumor purities. Somatic mutations were called using the muTect algorithm [8], with data from patient's blood cells used as a normal comparison against archival or fresh-frozen tumor tissue. Somatic insertions and deletions were called in tumour and normal samples using GATK HaplotypeCaller; somatic mutations were defined as variants

that were only found in the tumour and validated through manual curation. Genes with significant numbers of mutations across all NET samples were identified using the MutSig algorithm [9].

Copy number analysis

To call allele specific copy-number (ASCN) segments from our exome sequence data, we generated variant and copy number calls using Varscan v2.3.6 [10] and used these as input “Sequenza” v2.1.0 R package [11]. Copy number profiles were selected on the basis of high posterior probabilities for the given architecture, in addition to favoring stable copy-number profiles.

To generate ASCN segments using Affymetrix GenomeWide 6.0 arrays, pre-processing was done using the Affymetrix Power Tools (APT) v1.16.1 suite of tools. The quality of the raw data was assessed prior to using the birdseed v2 algorithm in apt-probeset-genotype for genotype calling and apt-probeset-summarize for normalization of signal intensities. Upon manual revision of purity/ploidy estimates, ASCN were called using HAPSEG v1.1.1 [12] and ABSOLUTE v1.0.6 [13].

Copy number signature

Entropy for each chromosome was calculated using either copy-number states (gain, loss, or neutral) or zygosity (LOH or heterozygous). The CN signature was then generated by calculating a co-occurrence matrix between the CN state with the lowest entropy per chromosome and all other CN states (gain, loss, neutral) on other chromosomes for each sample. To get an estimate of significance regarding these observed co-occurrences, we computed a null matrix by shuffling the CN states for each chromosome in a single sample 1000 times, repeated across all samples. Using an empirical cumulative density function fitted for the null distribution, we calculated how rare the co-occurrences between CN states are.

Purity and loss of heterozygosity estimation

A given samples’ zygosity can be represented by the aggregated allelic fraction of variants which is dependent on factors such as purity and ploidy. Identification of theoretical allelic fractions F that are representative of LOH are calculated as a function of the expected abundance of the Alt and Ref allele for the tumor $\mathtt{T}$ and normal $\mathtt{N}$ samples (tumor-alt A, tumor-ref R, normal-alt a, normal-ref r). For instance, a SNV affecting one of two alleles would have an F_T of 0.5 and F_N of 0. These estimated abundances are calculated in equation (1) as a product of the estimated ploidy P and theoretical allelic fractions for a given tumor sample:

$$A = \frac{F_T P_T}{2} \qquad (1)$$

$$a = \frac{F_N P_N}{2}$$

$$R = (1 - F_T)\frac{P_T}{2}$$
$$r = (1 - F_N)\frac{P_N}{2}$$

This approximation is used to estimate the purity-adjusted alternate and reference allele fractions for the tumour sample that should be seen in the data with the given parameters.

$$F_{Alt} = \frac{A(p_t) + a(1 - p_t)}{A(p_t) + a(1 - p_t) + R(p_T) + r(1 - p_T)} \qquad (2)$$
$$F_{Ref} = 1 - F_{Alt}$$

Given our pathologist-estimated purities for each tumour sample, we were able to calculate the alternate and reference allelic fraction to be representative of LOH. This was done by rearranging equation (2) to obtain purity and ploidy adjusted allelic fractions where a value of 1.0 indicates the presence of a mutant variant on all alleles:

$$F_T = \frac{F_{Alt}(P_N - P_N p_T + P_T p_T)}{P_T p_T} \qquad (3)$$

In order to establish a computationally-predicted purity, we approximated the allelic fraction that is representative of LOH by using a kernel density estimation (KDE) on CBS-smoothed allelic fractions. By attributing to each variant its CBS segmented allelic fraction, we minimize the noise-induced dispersion, thus allowing for more defined peaks in the KDE curves.

The two outermost KDE peak were considered representative of LOH allelic fraction densities for the reference and alternate allele in tumor samples. By rearranging equation (2), we can further estimate the tumor purity of a sample by taking the local maxima of the LOH peaks as an approximation for the alternate and reference allelic fractions.

$$p_T = \frac{a(F_{Alt} - 1) + r(F_{Alt})}{a(F_{Alt} - 1) - AF_{Alt} + A + F_{Alt}(r - R)} \qquad (4)$$

Allelic fractions of 0 and 1.0 to the immediately adjacent inner minimas for both peaks were taken as an approximate range for all LOH segmented allelic fractions. The densities of the raw variant allele fractions for each segment within the LOH range were manually inspected to ensure that they maintained a similar unimodal peak and that the segmented allelic fractions were representative. Final LOH segment approximation was taken as any segment below the 95th quantile for the lower reference allele peak and above the 5th quantile for the upper alternate allele peak.

Conversion of CGH cytogenetic data to genomic coordinates

CGH cytogenetic data was obtained from tables published in the literature [14–21] and transcribed into genomic coordinates by using the UCSC Table Browser hg19 cytoBandIdeo file as a

reference [22]. Each segment was given a value corresponding to the copy-status; a gain was given a value 1, a loss -1, and copy-neutral status was assigned a 0. The hg19 genome was then segmented into 100kb bins, based on the reported lower limit of resolution in array CGH [23], and the copy number status for each bin was given according to the intersecting segment in the sample. Bins that spanned breakpoints were weighted according to the fraction of the bin within each copy-number segment:

$$x_i = \frac{1}{S}\sum_{s=1}^{S}(w_s h_s) \qquad (5)$$

where x_i is the copy-number value for bin i, corresponding to the average of all copy number values h weighted by the fraction of the bin they occupy w for every copy number segment S within the bin range.

Asymmetric matching of CGH copy-number profiles

To quantify the degree of similarity between any two samples, we used the Jaccard index as a measure of asymmetric binary concordance; a robust statistic that is foundation of TumorComparer, a cell line-tumor similarity quantification tool [24]. This statistic accommodates for the large degree of the genome that does not have any copy-number aberration by removing copy-neutral matches between bins with the same genomic coordinates.

An all-by-all sample comparison was calculated as

$$S_{XY} = \frac{\sum_{i=1}^{n} m_i, m_i \begin{cases} 1 & (x_i = y_i)\ \&\ (x_i \neq 0) \\ 0 & (x_i \neq y_i) \end{cases}}{\sum_{i=1}^{n} 1 \mid (x_i \neq 0) \parallel (y_i \neq 0)} \qquad (6)$$

where S_{XY} is the Jaccard similarity coefficient given for samples X and Y; Samples are represented by feature vectors $X = (x_1, x_2, , x_n)$ and $Y = (y_1, y_2, , y_n)$. Each feature is compared and given a binary value, , according to whether the features from the two match and are non-zero.

Clustering and meta-analysis of CGH studies

Hierarchical agglomerative clustering using the euclidean distance between Jaccard similarity coefficients was used as an unsupervised metric to segregate the copy-number profiles. The genomic fraction of chromosomal instability (CI) was used as the best measure of separation to distinguish between cluster 2 from cluster 1,3, and 4. A cutoff threshold for high- and low-CI was determined by the minimum, non-zero, intersection of the kernel density functions for each of the 4 clusters. Association tests were carried out with the “vcd” v1.4-1 R package [25] to compute the Pearson’s chi-squared and Cramér’s V statistic on contingency tables for

high-/low-CI feature against metastasis status, NET type, study ID, and functionality of the tumor. A meta-analysis among the features listed was done using a random/mixed-effects models and tested with Cochrane's Q test, implemented in the "metafor" v1.9-8 R package [26].

PanCancer analysis

Allele-specific copy number data for the TCGA PanCancer cohort was obtained from the Carter et al. (2012) paper. Loss-of-heterozygosity and copy-states for each copy-number segment were taken directly from their published results and were used to estimate the fraction of the genome afflicted by the LOH state. The pediatric GIST and Rhabdoid tumour types were removed from this cohort on the basis of having fewer than 10 samples. Malignant fibrous histiocytoma (MFH) was also removed on the basis of it being an antiquated disease term that the World Health Organization declassified in 2002, renaming as undifferentiated pleomorphic sarcoma not otherwise specified (NOS) [27]. A 95% confidence interval was calculated for the LOH fraction for each remaining tumour type in addition to our discovery and validation cohort by fitting the data to a t-distribution.

PANET Cell Lines

Whole-exome sequencing of the BON-1 and QGP-1 PANET cell line from VanDamme and colleagues[28] was re-analyzed and LOH segments were called based on allelic fractions (European Nucleotide Archive study ID: PRJEB8223).

AACR GENIE Analysis

Copy-number profiles and mutational data of PANETs from AACRs project GENIE (v1.0.1) were downloaded from Sage Synapse (https://www.synapse.org/; synapse IDs: syn7851250, syn7851253, and syn7851246). In total, 43 PANET samples had both copy-number information and mutational information. The molecular timing of these samples was determined by using the computationally estimated allelic fraction for each SNV to estimate the tumour purity (Equation 4) for every possible copy-number state. For instance, an SNV with a 0.40 allelic fraction in a diploid tumor could correspond to a tumour purity of 0.80 or 0.40 depending on whether the SNV was heterozygous or homozygous respectively. We next added the constraint that the total copy-state for the SNV is dictated by the the estimated copy-ratio of that region (e.g. SNV_C has a log2-ratio of 1 compared to SNV_A and SNV_B which are 0. This indicates that $SNV_{A/B}$ can have a copy-state of 2 if SNV_C is a copy-state of 4.). We add another constraint that only models where all estimated purities for the SNVs must be within a 0.15 range of the pathologist purity. Finally, we select the best model that explains all SNVs and has the simplest copy-number profile (e.g., total copy-state is 2 instead of 8) to infer molecular timing of the disease. If the mutations happened prior to LOH and genome doubling, we would expect

to see the allelic fraction correspond to a model where all alleles in the tumour fraction are mutated.

Shallow WGS analysis

To detect copy-number and chromosomal instability in an extended validation cohort, we performed shallow ~0.3x PCR-free whole-genome sequencing beginning with an input of 100 ng of DNA from each tumour sample (Lucigen NxSeq AmpFREE Low DNA Library Kit). Estimation of copy number was achieved by calculating the physical coverage of paired-end 75 bp reads for each fragment (median ~405 bp insert size). By segmenting the genome into 500kB bins, we were able to count the number of fragments found within each bin. The aggregate coverage across the entire genome was modelled using a gaussian kernel density estimation with the Sheather & Jones (1991) method to better approximate the bandwidth [29]. Using the local maximas of each peak as a seed, we further decomposed the mixed distribution into a finite number of gaussian distribution models to represent copy-states. The parameters for these copy-state distribution models were estimated using the EM algorithm implemented in the R package mixdist v0.5-4. The observed fragment counts for each 500kB bin were then mapped against a binomial model fitted around the MLE estimation for each Gaussian distribution. Copy-number was then assigned based on the binomial model that yielded the highest probability for the observed bin fragment count. When available, FISH data was used to scale the copy-number fit accordingly.

Due to the limitations of estimating allelic fractions in shallow WGS, we employed an alternate method that identifies whether a given 500kB bin is deficient or enriched in heterozygous variants. MuTect v1.1.5 was run in tumor-only mode to force call all variants in the dbSNP build 146 database, disregarding all quality control metrics for the estimated variants. A heterozygous variant was defined as any variant that did not have an allelic fraction of 0 or 1. As a normal genome reference, we generated an empirical cumulative density function (ECDF) for the number of heterozygous variants found within a 500kB bin across the entire genome for all samples. For each tumor sample, we then counted the number of heterozygous variants within a given 500kB bin and converted that to a percentile in relation to the reference ECDF.

To estimate segments of LOH, we collapsed all consecutive genomic bins that maintained the same copy-state to generate segments of continuous copy-number aberrations. For each segment, we found the median percentile corresponding to the number of heterozygous variants within a 500kB bin. To assess whether pathologist-estimated stromal content would be a good a threshold to identify whether a bin was LOH or heterozygous, we used linear regression to model the relationship between stromal content and the median percentile of heterozygous variants across the entire chromosome in largely diploid cancer like NETs. Stromal content was determined to be a good metric for this threshold as the median percentile corresponding to heterozygous variants for a 500kB bin scaled linearly with stromal content. Using maximum likelihood estimation, we then selected the most prevalent percentile across each chromosome. A chromosome was termed as either LOH or heterozygous based on whether the

MLE-estimated percentile fell below the pathologist-estimated stromal content for that sample. Using the metafor R package, we then calculated the log odds-ratio between heterozygous variant-deficient and enriched chromosomes across different groups.

To estimate telomere length, cocleaned BAM files from shallow WGS were ran in telomerecat v3.1.2 using default parameters. Significance between GINETs and PANETs was obtained using a one-sided t-test.

FISH Analysis

Paraffin curls were deparaffinized in 3 sequential washes of 100% xylene. Extracted nuclei were re-hydrated in an alcohol series (95%, 75%, 50%) at 3 minutes per wash. Residual nuclei were mechanically disaggregated using a microtube pestle. Nuclei were then resuspended in 200µL of TE buffer to which 1µL of 20mg/mL Proteinase K was added. Solution is vortexed and placed in a 37C water bath for 20 minutes with one additional vortex at the 10-minute mark. After digestion, nuclei were microfuged at maximum speed for 10 minutes and Proteinase K solution was removed with a micropipettor leaving a small amount of supernatant as to not disturb the pellet. 200µL of Carnoy's fixative (3:1 methanol:acetic acid) was added and the tube was re-spun at max speed in a microcentrifuge for 10 minutes. Carnoy's was removed and process repeated two additional times. Fresh Carnoy's was then added in a volume relative to the pellet size to achieve an optimal density for FISH (i.e. 100-200µL).

15µL of isolated nuclei suspension was dropped on a positively charged slide. Slides were then air-dried. After checking for appropriate density slides were then baked for 60 minutes at 60C in a conventional oven. After baking, slides were immersed in 10mM sodium citrate buffer (pH6) for 2 hours at 80C. Slides were then rinsed in distilled water for 2 minutes followed by immersion in 2x SSC buffer for 5 minutes. Excess liquid was removed from the slide by blotting edges with a paper towel. Slides were then treated in pepsin solution for 15 minutes and washed in distilled water for 5 minutes. Slides were once again immersed in 2x SSC for 5 minutes. After pepsinization, slides were dehydrated in an ethanol series (70%, 90%, 100%) and allowed to air dry for 5 minutes. Slides were then hybridized with FISH probe(s) (as per manufacturer's instructions), denatured on a Thermobrite at 75C for two minutes and hybridized overnight at 37C in a humidified chamber. Slides are then washed following the manufacturer's instructions for specific probes and mounted with 10uL of DAPI solution and mounting medium (100µL DAPI:900µL mounting medium).

CENP-A ChIP analysis

WIG files for the Nechemia dataset were downloaded from GEO:GSE111381 [44], while bigwig files for the Nye dataset were downloaded from GEO:GSE120230 [35] and converted to WIG files using the UCSC tool bigwigToBedgraph. Peaks were assigned to cytobands based on the hg19

reference genome. Nye et al. originally defined a “merged peaks” representation of their *DAXX* and control group by overlapping the peaks for all biological replicates and taking the min and max interval for each overlap. We summarized CENP-A peak intensities across each merged peak by using two complementing metrics: the max peak height for each merged peak, or reads per kilobase of peaks per million mapped reads (RPKM).

For the Nye dataset, we compared all overlapping peaks between *DAXX* and control groups using a t-statistic. We then summarized the t-statistic of all overlapping peaks for each cytoband by taking the mean and standard deviation. Similarly, by including all peaks in *DAXX* or control groups in each cytoband, we calculated a Kolmorogov-Smirnov D-statistic by comparing the peaks found only in that cytoband against peaks found across the entire genome. This D-statistic was averaged across all biological replicates.

Centromeric gene expression analysis

We analyzed 148 PANET gene expression profiles obtained from published microarray datasets: 99 generated by Sadanandam et al. (GSE73338) and 49 from Chan et al. (GSE117851). To approximate whether the CN-signature is retained in MAD+ PANETs from these datasets, we first separated samples based on whether they carried MAD mutations and computed the z-score for gene expression against the MAD- PANETs on a per gene basis. Genes were mapped back to the human genome assembly hg19 and a loess regression with a 50% smoothing span was fitted to these values.

For each chromosome arm, we calculated the calculated the cumulative density function (CDF) for all expressed genes on that arm. Using this CDF, we assigned a percentile to each gene. After collapsing all chromosomes down into two groups (LOH and Het), we calculated the chromosome arm-specific fractional distance to the centromere. Using the distance to the centromere and expression quantile values, we fit a loess regression with a 10% smoothing span. The distance between these two loess regression curves was calculated at 0.01 bins. We also estimated a null distribution of distances for each bin by randomly assigning chromosomes to LOH or Het group and recomputing the distance between curves 1000 times. Finally, we estimated the p-value by calculating the z-score of our observed distance from the null distribution at each 0.01 bin.

References

1. Peña-Llopis, S. & Brugarolas, J. Simultaneous isolation of high-quality DNA, RNA, miRNA and proteins from tissues for genomic applications. Nat. Protoc. 8, 2240–2255 (2013).
2. Li, H. & Durbin, R. Fast and accurate long-read alignment with Burrows–Wheeler transform. Bioinformatics 26, 589–595 (2010).
3. Dobin, A. et al. STAR: ultrafast universal RNA-seq aligner. Bioinformatics 29, 15–21 (2013).
4. DePristo, M. A. et al. A framework for variation discovery and genotyping using next-generation DNA sequencing data. Nat. Genet. 43, 491–498 (2011).
5. Van der Auwera, G. A. et al. From FastQ data to high-confidence variant calls: the genome analysis toolkit best practices pipeline. Curr. Protoc. Bioinformatics 11–10 (2013).
6. McKenna, A. et al. The Genome Analysis Toolkit: a MapReduce framework for analyzing next-generation DNA sequencing data. Genome Res. 20, 1297–1303 (2010).
7. Olshen, A. B., Venkatraman, E. S., Lucito, R. & Wigler, M. Circular binary segmentation for the analysis of array-based DNA copy number data. Biostatistics 5, 557–572 (2004).
8. Cibulskis, K. et al. Sensitive detection of somatic point mutations in impure and heterogeneous cancer samples. Nat. Biotechnol. 31, 213–219 (2013).
9. Lawrence, M. S. et al. Mutational heterogeneity in cancer and the search for new cancer-associated genes. Nature 499, 214–218 (2013).
10. Koboldt, D. C. et al. VarScan 2: somatic mutation and copy number alteration discovery in cancer by exome sequencing. Genome Res. 22, 568–576 (2012).
11. Favero, F. et al. Sequenza: allele-specific copy number and mutation profiles from tumor sequencing data. Ann. Oncol. 26, 64–70 (2015).
12. Carter, S. L., Meyerson, M. & Getz, G. Accurate estimation of homologue-specific DNA concentration-ratios in cancer samples allows long-range haplotyping. Nat. Preced 59–87 (2011).
13. Carter, S. L. et al. Absolute quantification of somatic DNA alterations in human cancer. Nat. Biotechnol. 30, 413–421 (2012).
14. Zikusoka, M. N., Kidd, M., Eick, G., Latich, I. & Modlin, I. M. The molecular genetics of gastroenteropancreatic neuroendocrine tumors. Cancer 104, 2292–2309 (2005).
15. Terris, B. et al. Comparative genomic hybridization analysis of sporadic neuroendocrine tumors of the digestive system. Genes Chromosomes Cancer 22, 50–56 (1998).
16. Kulke, M. H. et al. High-resolution analysis of genetic alterations in small bowel carcinoid tumors reveals areas of recurrent amplification and loss. Genes Chromosomes Cancer 47, 591–603 (2008).
17. Stumpf, E. et al. Chromosomal alterations in human pancreatic endocrine tumors. Genes Chromosomes Cancer 29, 83–87 (2000).
18. Tönnies, H. et al. Analysis of sporadic neuroendocrine tumours of the enteropancreatic system by comparative genomic hybridisation. Gut 48, 536–541 (2001).
19. Speel, E. J. M. et al. Genetic Evidence for Early Divergence of Small Functioning and Nonfunctioning Endocrine Pancreatic Tumors. Cancer Res. 61, 5186–5192 (2001).

20. Zhao, J. et al. Genomic imbalances in the progression of endocrine pancreatic tumors. Genes Chromosomes Cancer 32, 364–372 (2001).
21. Floridia, G. et al. Chromosomal alterations detected by comparative genomic hybridization in nonfunctioning endocrine pancreatic tumors. Cancer Genet. Cytogenet. 156, 23–30 (2005).
22. Karolchik, D. et al. The UCSC Table Browser data retrieval tool. Nucleic Acids Res. 32, D493–6 (2004).
23. de Ravel, T. J. L., Devriendt, K., Fryns, J.-P. & Vermeesch, J. R. What's new in karyotyping? The move towards array comparative genomic hybridisation (CGH). Eur. J. Pediatr. 166, 637–643 (2007).
24. Sinha, R., Schultz, N. & Sander, C. Comparing cancer cell lines and tumor samples by genomic profiles. bioRxiv 028159 (2015). doi:10.1101/028159
25. Meyer, D. et al. Package 'vcd'. (2017).
26. Viechtbauer, W. & Others. Conducting meta-analyses in R with the metafor package. J. Stat. Softw. 36, 1–48 (2010).
27. Fletcher, C. D. M., Krishnan Unni, K. & Mertens, F. Pathology and Genetics of Tumours of Soft Tissue and Bone. (IARC, 2002).
28. Vandamme, T. et al. Whole-exome characterization of pancreatic neuroendocrine tumor cell lines BON-1 and QGP-1. J. Mol. Endocrinol. 54, 137–147 (2015).
29. Sheather, S. J. & Jones, M. C. A Reliable Data-Based Bandwidth Selection Method for Kernel Density Estimation. J. R. Stat. Soc. Series B Stat. Methodol. 53, 683–690 (1991).
30. Vogelstein, B. et al. Cancer genome landscapes. Science 339, 1546–1558 (2013).
