# Supplementary material for: Centromeric cohesion failure invokes a conserved choreography of chromosomal mis-segregations in pancreatic neuroendocrine tumor
Source: Genome Med. 2020 Apr 28;12:38. doi: 10.1186/s13073-020-00730-9 (PMC7189550; doi:10.1186/s13073-020-00730-9)
Supplement: Supplementary file 3 — Additional file 3. Supplementary figures for the manuscript. [file 13073_2020_730_MOESM3_ESM.pdf]

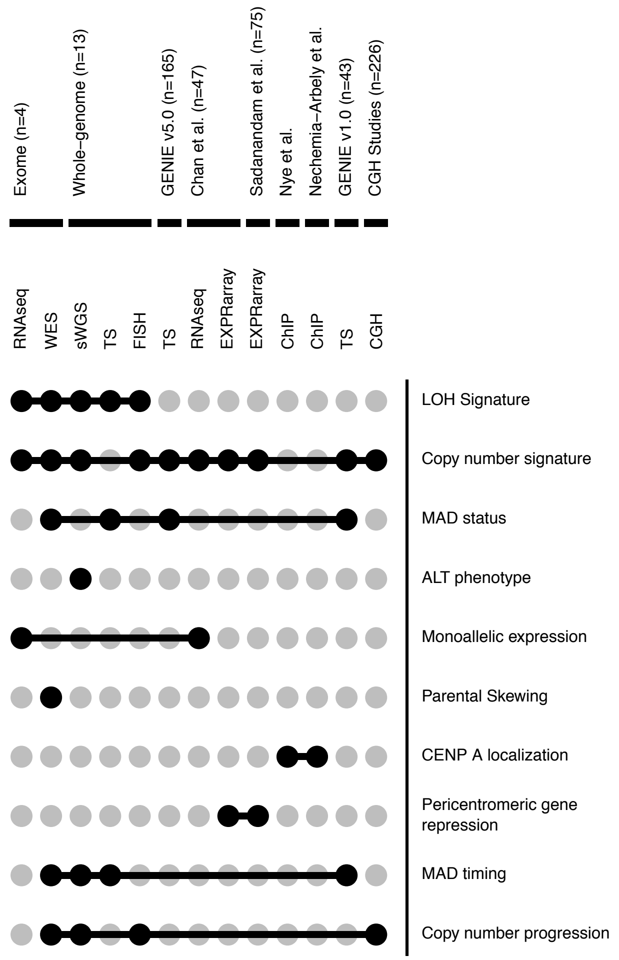

Figure S1

Mapping of datasets used to analysis performed. The identification and size of the dataset is mapped to the platforms used with a black line. The platforms are either RNA sequencing (RNAseq), whole-exome sequencing (WES), shallow whole-genome sequencing (sWGS), fluorescence in situ hybridization (FISH), targeted sequencing (TS), expression array (EXPRarray) or comparative genomic hybridization (CGH).

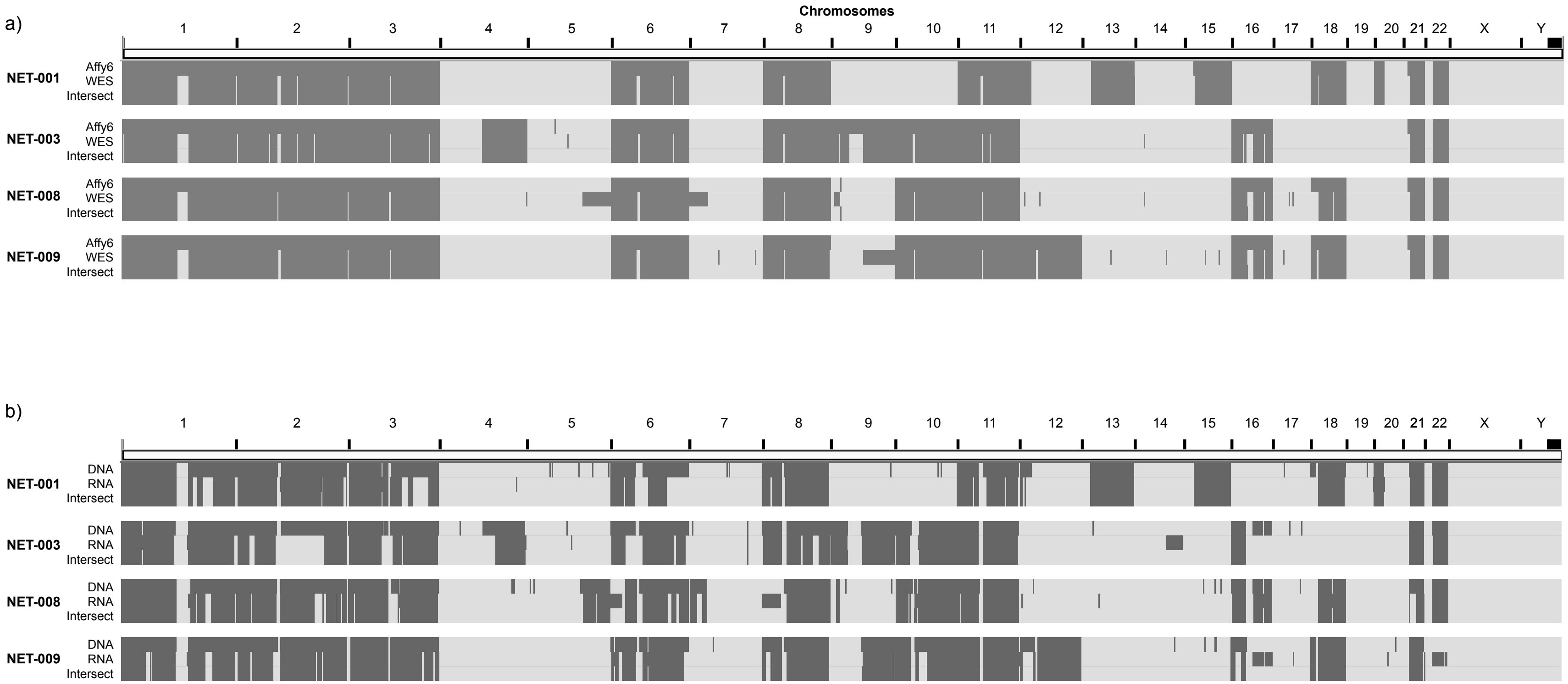

Figure S2

Loss of heterozygosity regions found within the same sample using two different technologies. a) LOH regions identified across the genome for the Genome-wide Human SNP Array 6.0 platform (Affy6) and the sample-matched whole-exome sequencing (WES) platform, as well as their intersect. b) LOH regions identified across the genome for the DNA (whole-exome sequencing) and RNA (RNA-sequencing), as well their intersect.

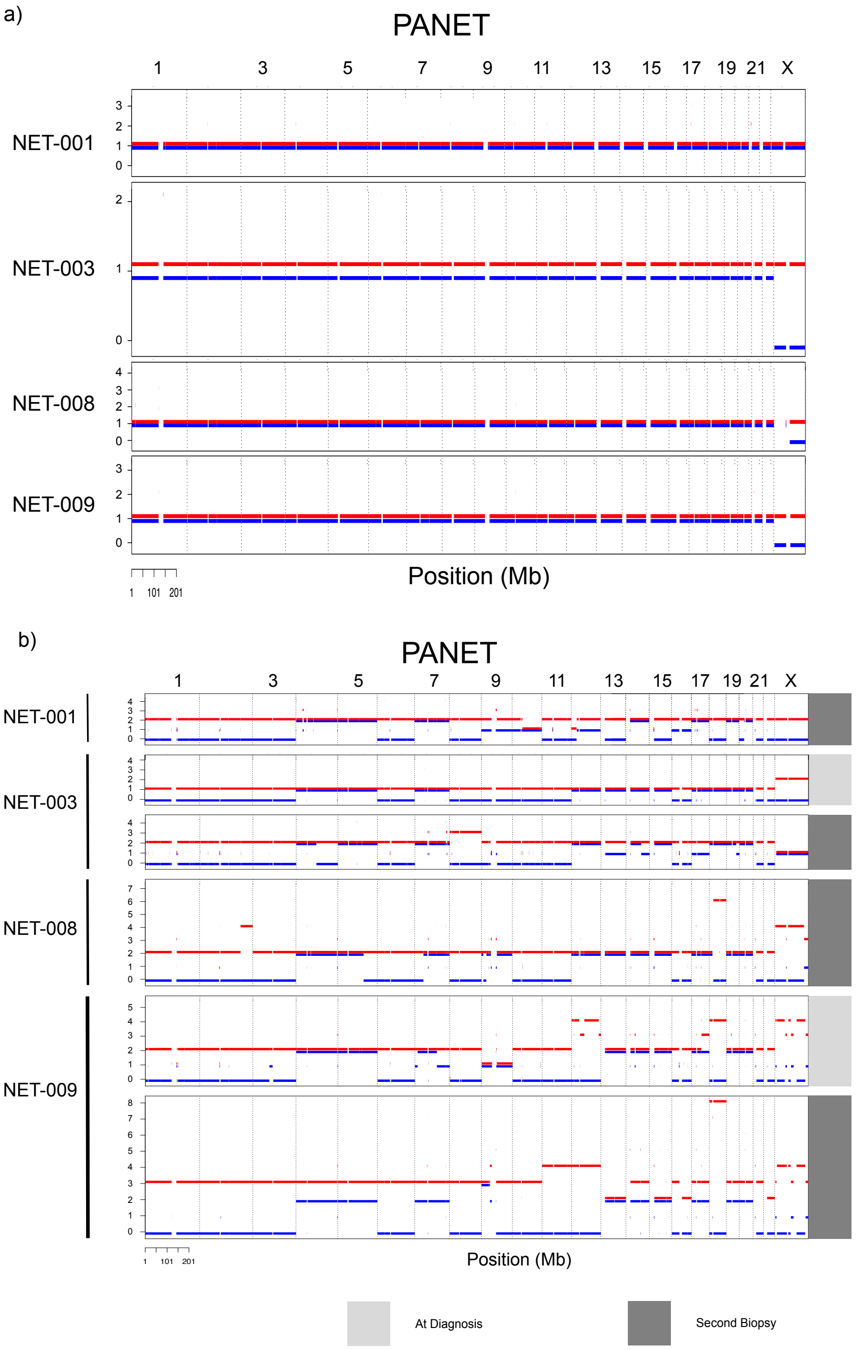

Figure S3

Allele specific copy-number plots for each tumour type in the discovery cohort. a) Copy number plots estimated from the Varscan2 and Sequenza algorithm for whole-exome sequenced samples. b) Copy number plots estimated for the "second biopsy" samples from the HAPSEG and ABSOLUTE algorithm for Genome-Wide Human SNP Array 6.0.

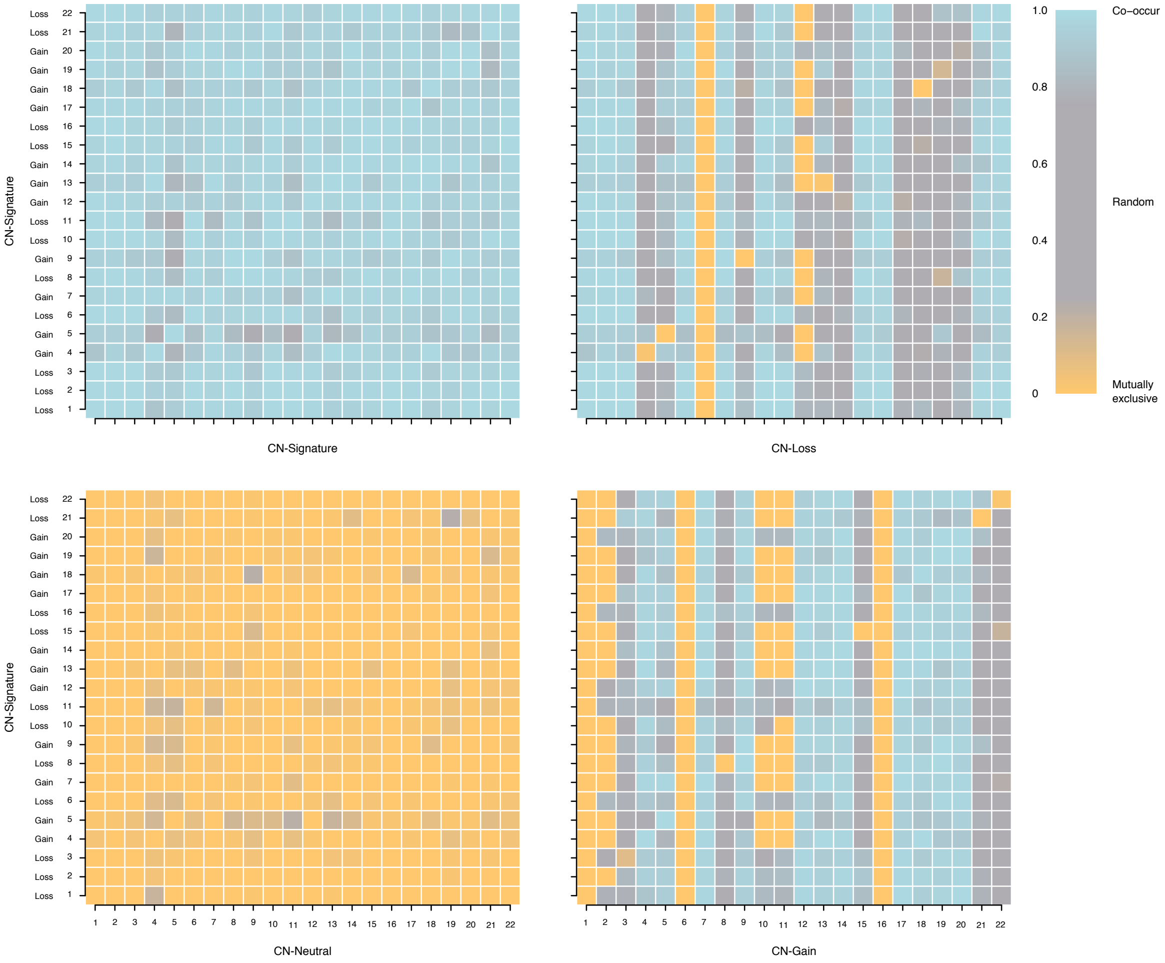

Figure S4

Co-occurrence plots between the most prominent CN states (y-axis) plotted against itself, loss of all chromosomes (log2 <= -0.2), neutral for all chromosomes (-0.2 < log2 < 0.2), or gain (log2 >= 0.2). Colours show a continuous scale of negative (orange) to positive selection (blue) for co-occurence when compared against a null distribution composed of randomizing and resampling single-sample copy number profiles 1000 times.

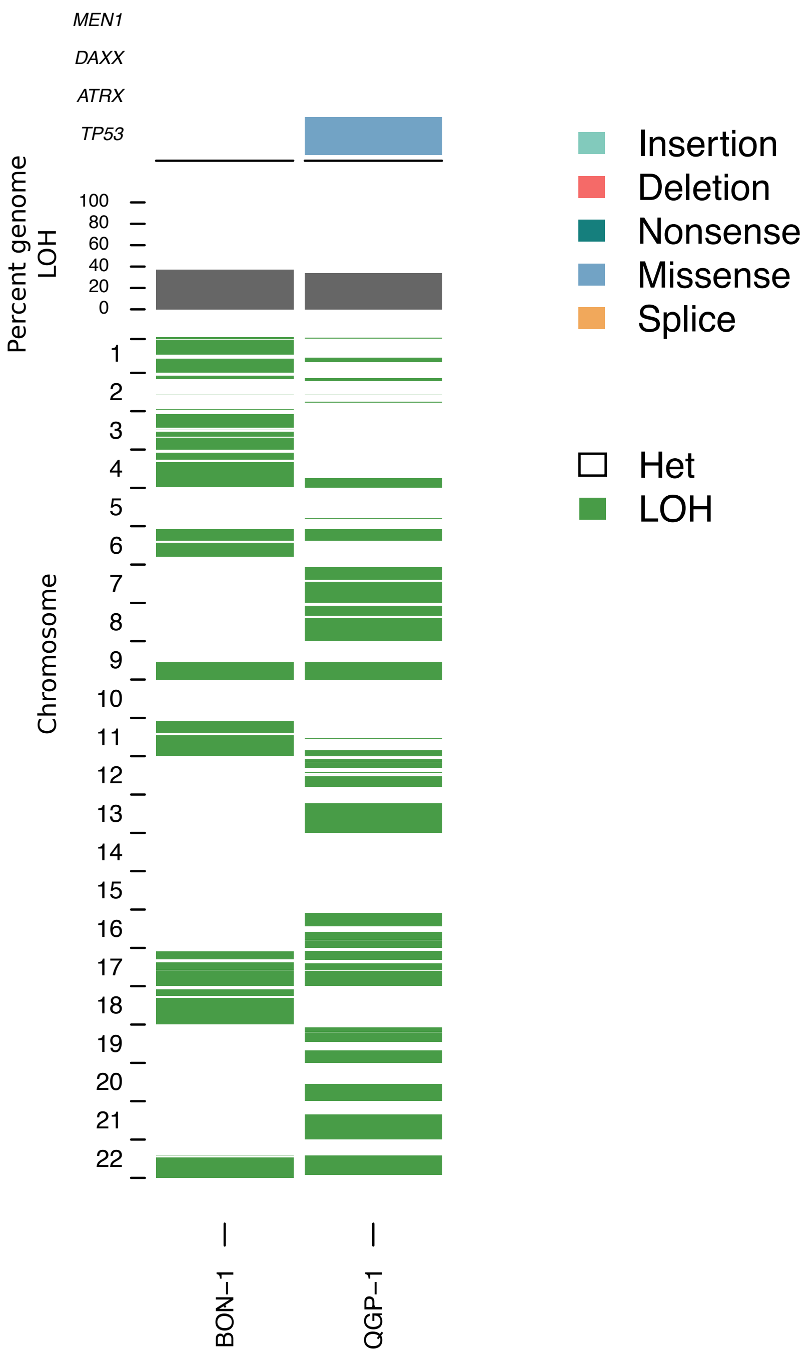

Figure S5

Zygosity plot of PANET model systems BON-1 and QGP-1. The green colour indicates an unknown copy-state but the presence of LOH.

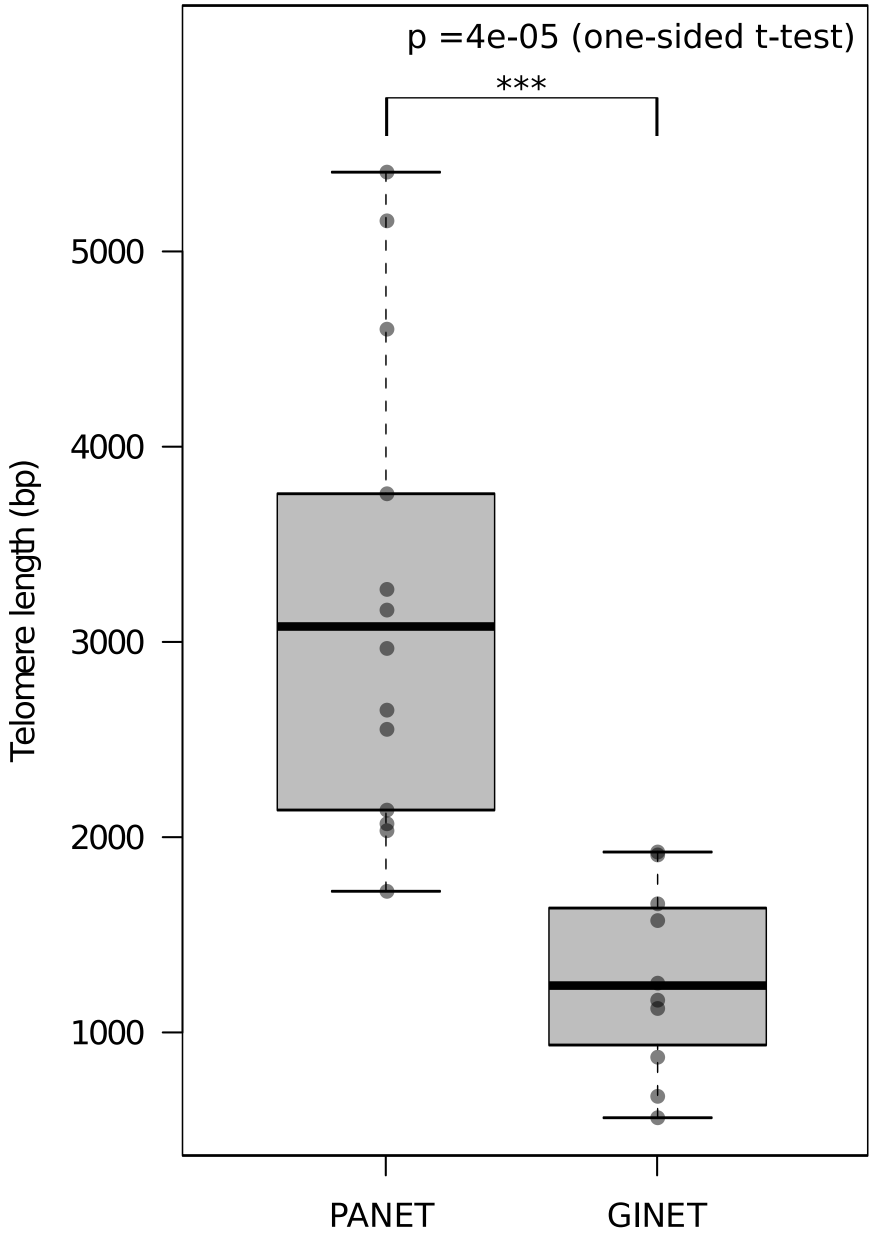

Figure S6

Estimated telomere length between pancreatic NETs and gastrointestinal NETs sequenced using shallow whole-genome sequencing. All samples in the PANET cohort are MAD+ with the exception of a single MAD- sample, NET-129 (red). A one-sided t-test was used to calculate statistical significance between telomere lengths.

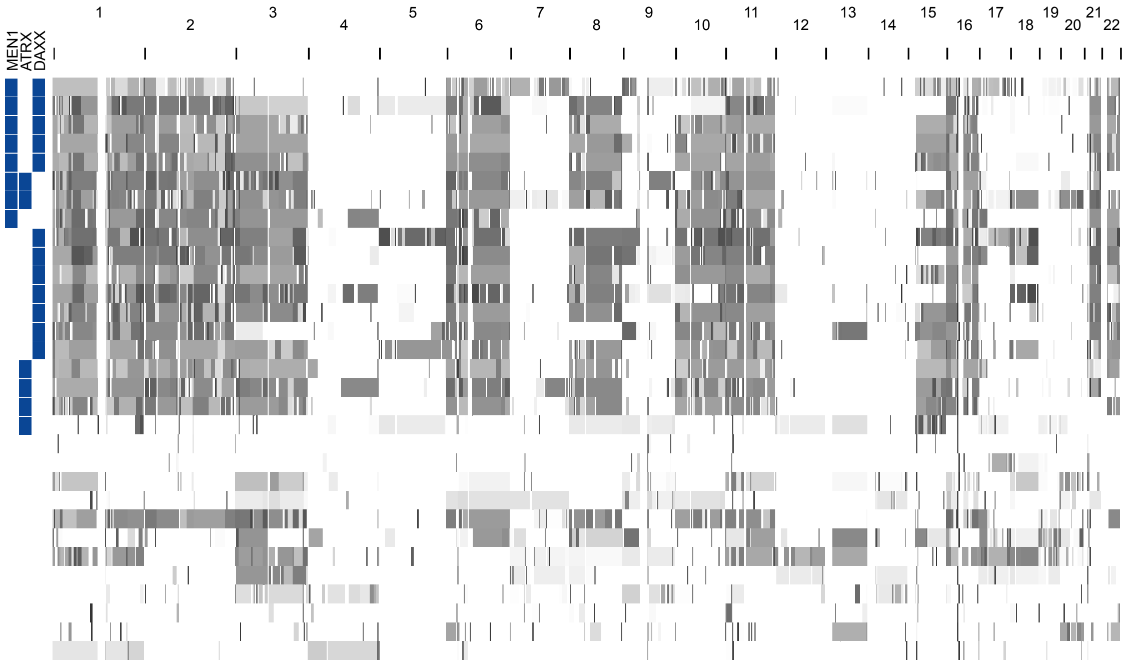

Figure S7

LOH and CN-signature of the Chan et al. dataset as represented through expression data. a) Allelic fractions from SNPs called on RNAseq data for each sample, annotated with mutation in MEN1, ATRX or DAXX. Values range from 0 to 0.5 with darker shades (~0) indicated homozygous SNPs and white indicating heterozygous (~0.5).

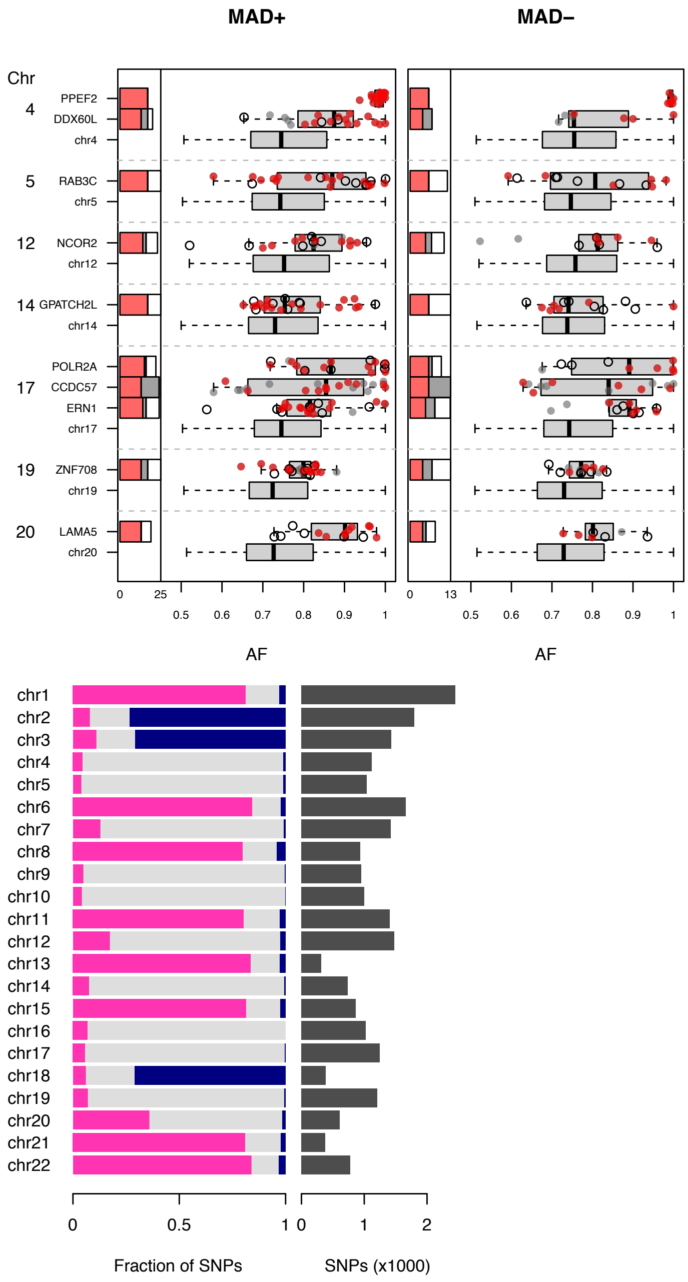

Figure S8

Deciphering the selection mechanism underlying recurrent LOH chromosomes. **a)** Monoallelic expression of genes significant in MAD+ PANETs. Boxplots represent the average alternate allelic fraction (AF) for all SNPs in that sample per gene. Points are coloured based on whether the samples had significant MAE ($q < 0.2$) (red), non-significant MAE ($q >= 0.2$) (grey), or lacked enough SNPs to test (white). The number of samples included in the analysis are represented as a stacked barplot. **b)** Parental skewing of SNPs on LOH chromosomes. The right panel is the number of SNPs that were homozygous in the maternal DNA and heterozygous in the non-tumour DNA of the offspring (NET-001). The left panel is the fraction of these homozygous to heterozygous SNPs in the tumour DNA of the offspring that were also heterozygous (grey), or if they matched to either the maternal (pink) or paternal (blue) DNA.

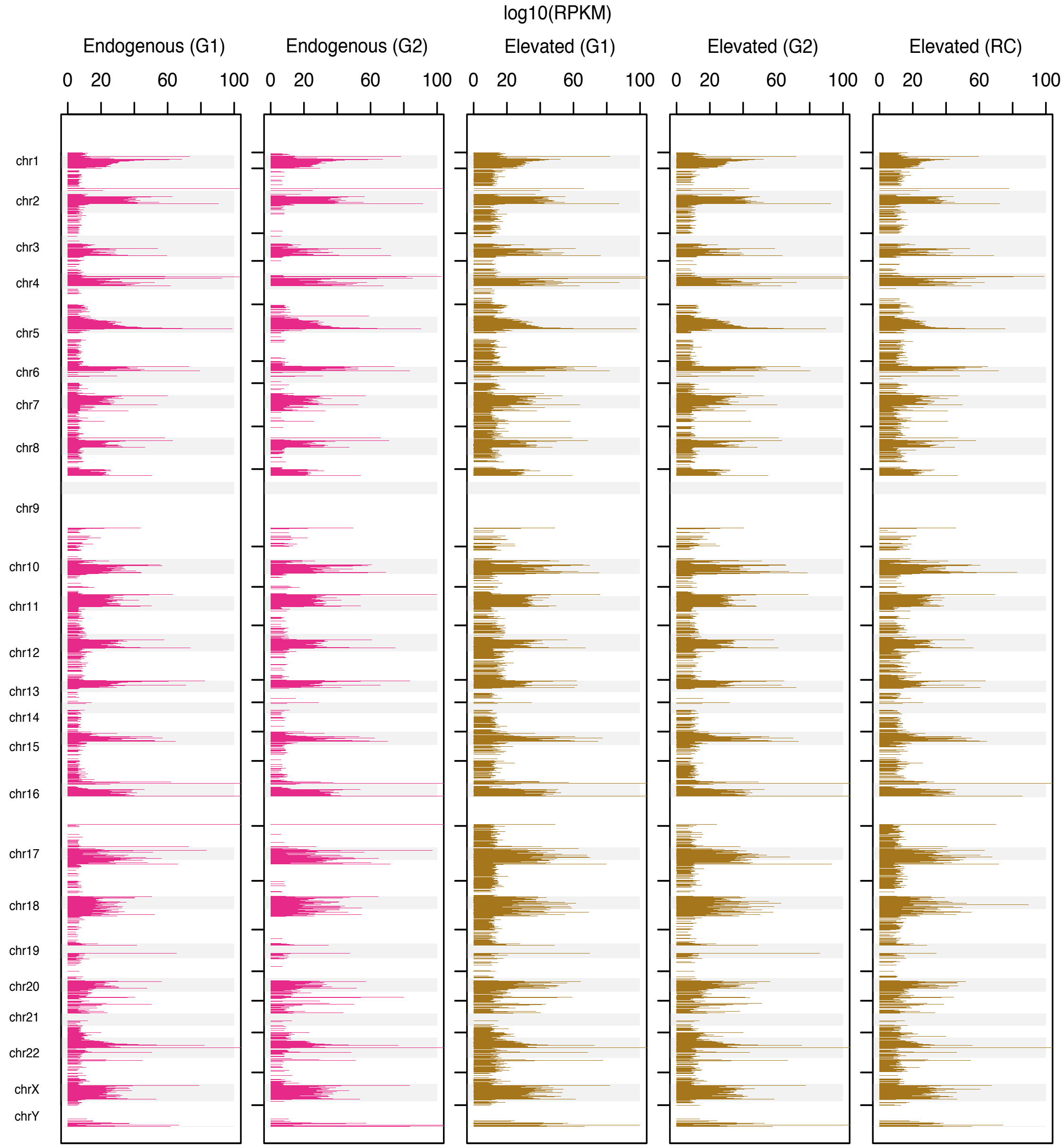

Figure S9

Peak heights for CENP-A at different stages of cell cycle for endogenous or elevated expression. Data is obtained from the Nechemia et al. dataset.

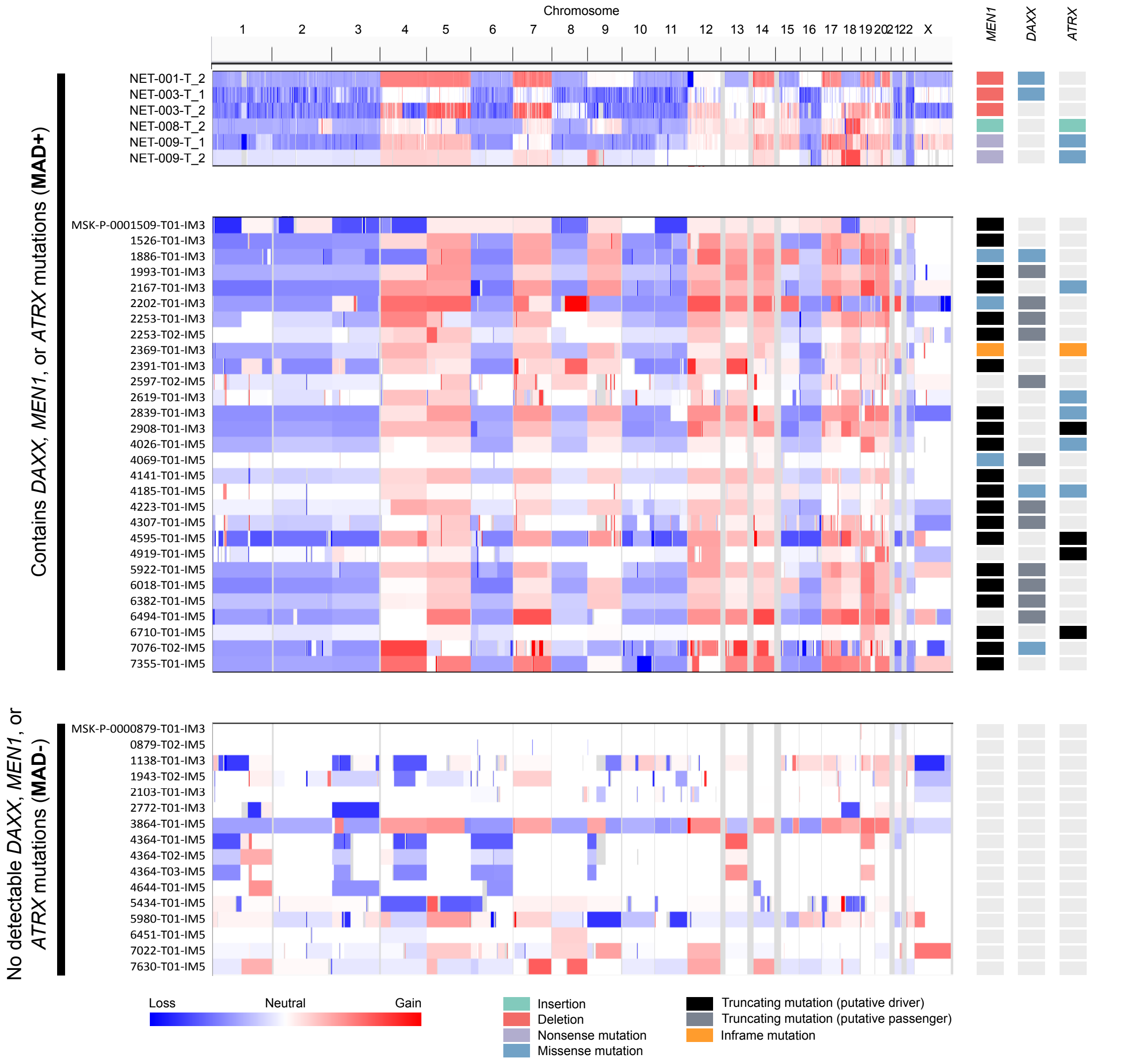

Figure S10

Relative copy-number profiles for PANETs from the exome and GENIE cohorts. Samples are divided based on the presence of mutations on *MEN1*, *ATRX* or *DAXX* (MAD+), or the complete absence of mutations on any of these genes (MAD-)

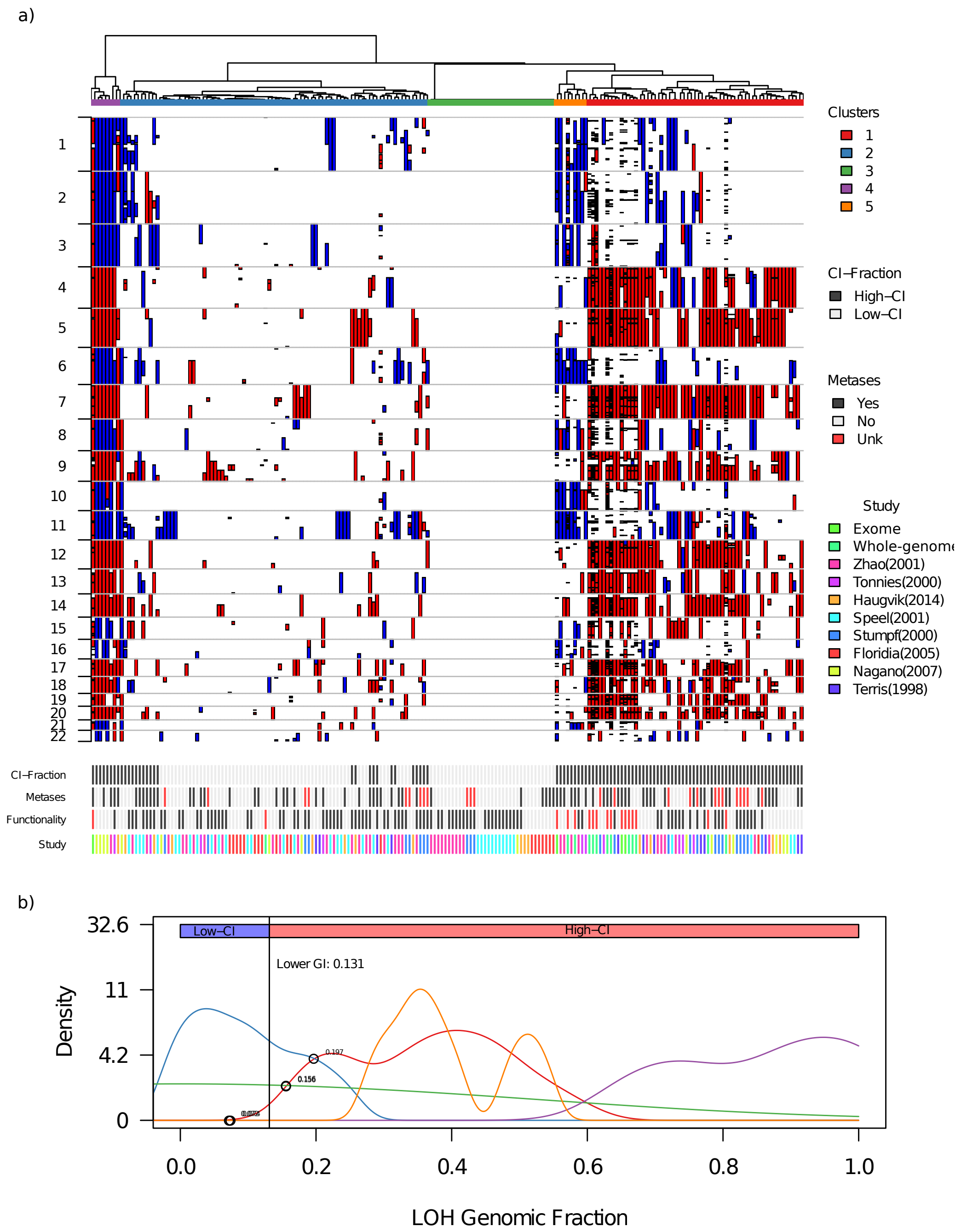

Figure S11

Hierarchical clustering of total copy number profiles from published CGH datasets with our discovery and validation cohorts. **a)** The corresponding ploidy for each chromosome is plotted as either a loss (1N: Blue) or gain (3N+: Red). The top track indicates hierarchical clustering based on the Jaccard coefficients between any two TCN profiles. The lower fractions represents annotations of the sample based on information mined from their respective studies. **b)** Kernel density profiles for the total genomic fraction aberrated in each cluster. The line separating the transition from “Low-CI” to “High-CI” is indicated as the average minimum intersection between density profiles of all clusters.
